# Regional variations in relative sea-level changes influenced by nonlinear vertical land motion

**DOI:** 10.1038/s41561-023-01357-2

**Published:** 2024-02-12

**Authors:** Julius Oelsmann, Marta Marcos, Marcello Passaro, Laura Sanchez, Denise Dettmering, Sönke Dangendorf, Florian Seitz

**Affiliations:** 1 Deutsches Geodätisches Forschungsinstitut, Technische Universität München (DGFI-TUM), Munich, Germany; 2 IMEDEA, (UIB-CSIC), Esporles, Spain; 3 Department of Physics, University of the Balearic Islands, Palma, Spain; 4 River-Coastal Science and Engineering, Tulane University, New Orleans, LA USA

**Keywords:** Projection and prediction, Ocean sciences, Solid Earth sciences, Climate-change impacts, Attribution

## Abstract

Vertical land movements can cause regional relative sea-level changes to differ substantially from climate-driven absolute sea-level changes. Whereas absolute sea level has been accurately monitored by satellite altimetry since 1992, there are limited observations of vertical land motion. Vertical land motion is generally modelled as a linear process, despite some evidence of nonlinear motion associated with tectonic activity, changes in surface loading or groundwater extraction. As a result, the temporal evolution of vertical land motion, and its contribution to projected sea-level rise and its uncertainty, remains unresolved. Here we generate a probabilistic vertical land motion reconstruction from 1995 to 2020 to determine the impact of regional-scale and nonlinear vertical land motion on relative sea-level projections up to 2150. We show that regional variations in projected coastal sea-level changes are equally influenced by vertical land motion and climate-driven processes, with vertical land motion driving relative sea-level changes of up to 50 cm by 2150. Accounting for nonlinear vertical land motion increases the uncertainty in projections by up to 1 m on a regional scale. Our results highlight the uncertainty in future coastal impacts and demonstrate the importance of including nonlinear vertical land motions in sea-level change projections.

## Abstract (web summary)

A probabilistic reconstruction of vertical land motion reveals regional variations in relative sea-level changes and large uncertainties in sea-level projections due to nonlinear effects.

## Main

Global mean absolute sea-level (GMSL) change has been increasing from rates of 1.4 mm yr^−1^ over the twentieth century to 3.25 mm yr^−^^1^ over the past decades (1993–2018) and is expected to accelerate further to rates of 5.2–12.1 mm yr^−1^ (during 2080–2100^1^). Coastal subsidence and uplift substantially modify the regional impacts of sea-level rise, generating signals in the order of 1–10 mm yr^−1^, which is similar to the regional variations of absolute sea-level change itself^2–4^. Accurate estimates of coastal vertical land motion (VLM) are thus needed to quantify future regional sea-level changes and associated socio-economic consequences.

However, several factors hamper the accurate estimation of VLM and its predictability along the world’s coastlines. Direct VLM observations (for example, by Global Navigation Satellite Systems; GNSS) are inhomogeneously distributed in space, often incomplete in time, and can be affected by several error sources such as equipment changes or platform settlement. The superposition of long-term processes such as the glacial isostatic adjustment (GIA), tectonic activity^5^, surface mass loading changes^6,7^ and other local natural or anthropogenic effects^8–10^ further complicates the assessment of the spatio-temporal characteristics of VLM. Despite this wide spectrum of VLM processes, previous research focusing on past sea-level changes and on sea-level projections for the forthcoming decades^1,11–13^ often implemented simplified assumptions about VLM. The current assumption is that VLM can be modelled as a linear process and as such projected into the future, which has been increasingly challenged in recent years^14,15^. In this study, we demonstrate that this assumption is not valid and show that VLM can introduce substantial uncertainties of up to 1 m in relative sea-level (RSL) change projections in 2150.

Previous sea-level projection studies so far mostly incorporated GIA models (for example, refs. ^16–18^) or indirect VLM estimates from long tide gauge records^19^ (as in the Sixth Assessment Report (AR6) of the Intergovernmental Panel on Climate Change (IPCC)^1^). However, the poor geographic coverage of tide gauges used and the lack of direct constraints, for example, GNSS measurements, which are the most precise and accurate technique for VLM determination, present some limitations of this dataset. More recent studies that have exploited existing networks of GNSS stations, tide gauges and altimetry data^4,20–22^ thus provide more robust interpolated VLM estimates. Notwithstanding the evidences of nonlinear VLM, these studies—including the estimates applied in sea-level projections—are based on the assumption of purely linear VLM. Therefore, we have only limited knowledge about the temporal variability of VLM, which impedes meaningful projections of VLM in areas where these variations are dominant.

**Fig. 1:**
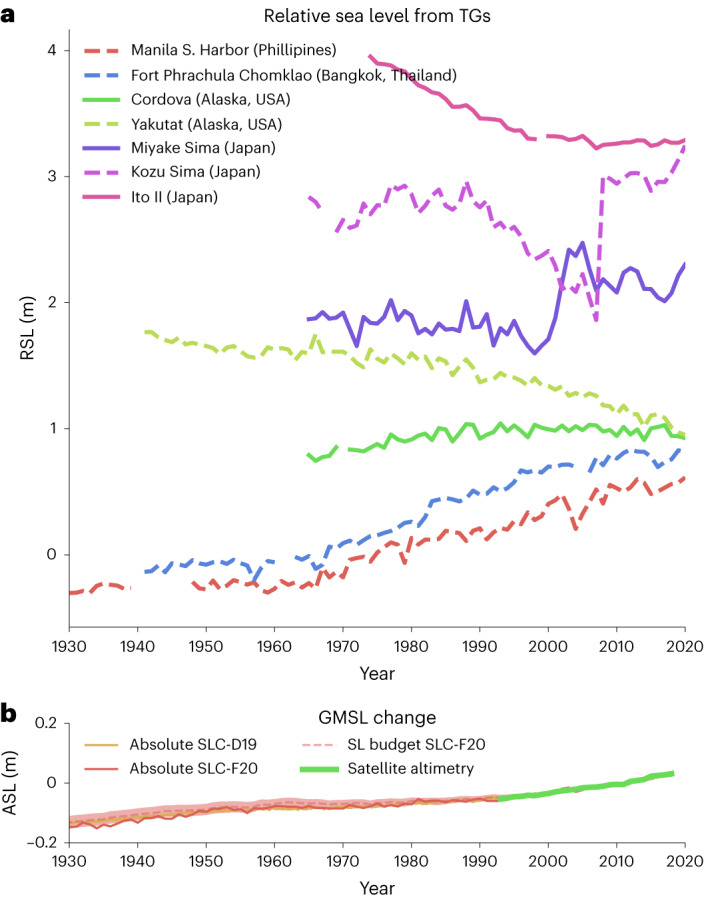
Historic sea-level changes. **a**, Long relative sea-level records from annually averaged tide gauge (TG) data. **b**, The global mean sea-level (SL) change from refs. ^13,22^. Satellite altimetry (Methods) and an estimate of the sea-level budget^22^.

Nonlinear VLM is not only present in current GNSS data, but it has also affected relative sea-level data from long tide gauge records (Fig. 1). Whereas tectonic activity can be associated with long-term VLM on timescales from hundreds of thousands to millions of years^23–25^, earthquakes often lead to instantaneous vertical displacements (as shown by the tide gauge in Kozu Sima), which are in many cases followed by postseismic decays^26–28^. Such nonlinear motion can hamper the determination of the inter-seismic trends, which is necessary for VLM projections into the upcoming centuries, as these rates can sustain from decades to centuries between earthquake events^29,30^. Several other processes such as contemporary mass changes (CMR^6,7,16^), erosion^31^, human-induced extraction of groundwater, oil and gas^8,10,32,33^, sediment loading and compaction^34,35^ and volcanism (tide gauge record at Miyake Sima in Fig. 1a) can also produce highly localized and nonlinear VLM. As an example, human-induced subsidence caused a strong acceleration in RSL around 1960 in Manila and Bangkok, leading to an overall RSL change of 75 cm since the beginning of the records, a change about four times as high as the GMSL change. Such high subsidence rates are problematic for many other large coastal cities^36^ and because of their nonlinear nature, introduce large uncertainties in future projections of the contribution of VLM to relative sea-level change.

Hence, to account for the non-GIA and nonlinear effects on VLM, we develop a new methodology to generate a time- and space-resolving VLM reconstruction for the global coastlines. We use this knowledge of the rates and temporal variations of VLM to assess the regional-scale impact of VLM on sea-level changes over the twentieth century and on projected (up to 2150) coastal RSL change and its associated uncertainties.

## Nonlinear VLM along global coastlines

**Extended Data Fig. 1:**
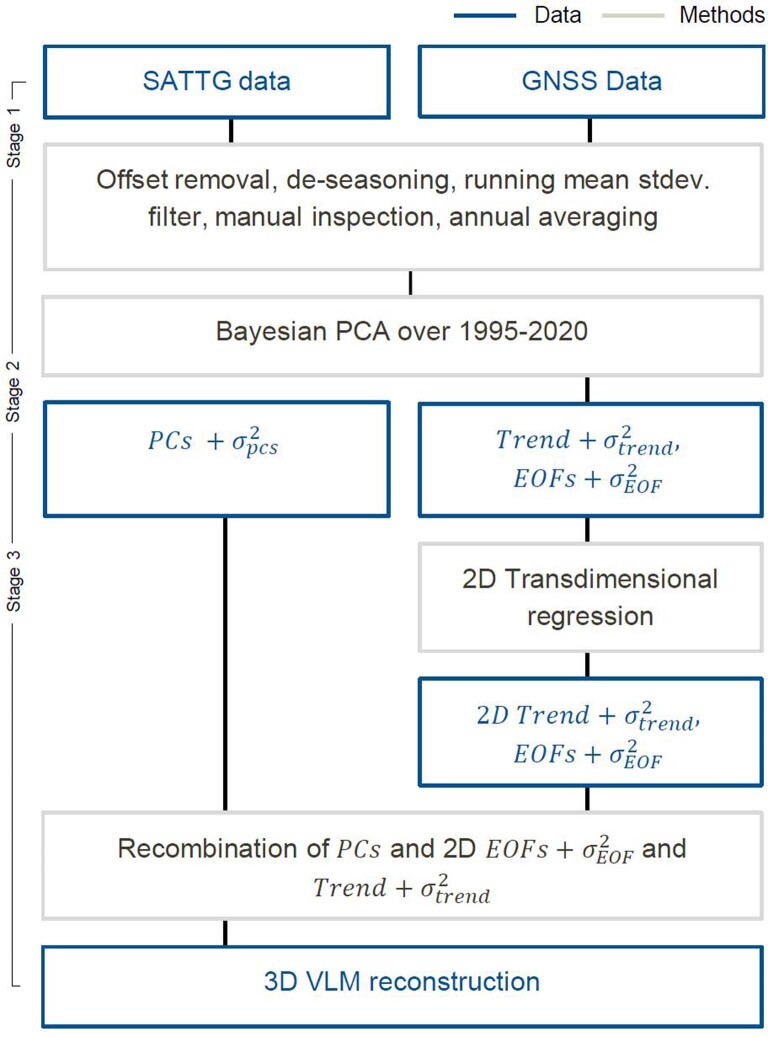
Overview of the data processing steps. Workflow describing the processing steps of generation of the vertical land motion (VLM) reconstruction dataset.

**Extended Data Fig. 2:**
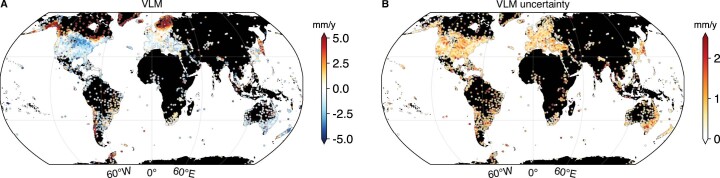
Vertical land motion (VLM) observations. Shown are VLM point estimates (**a**) and uncertainties (**b**, 95% CI) from a linear least squares fit.

**Extended Data Fig. 3:**
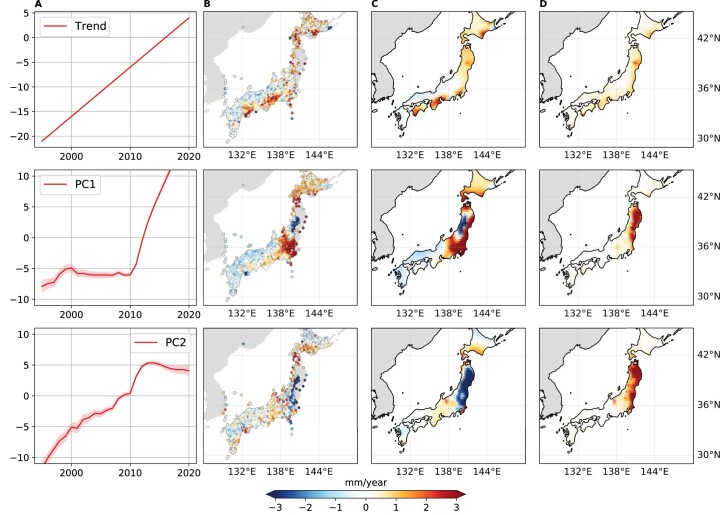
Spatio-temporal vertical land motion interpolation. The first column (**a**) shows the trend coefficient (top) and the first (middle) and second (bottom) principal components (and 1*σ* uncertainties) estimated for Japan [year]. **b** shows the corresponding point-wise spatial trend and EOF pattern. **c** displays the interpolated estimates of the pattern; the associated uncertainties are provided by **d**.

We reconstruct coastal VLM based on the joint probabilistic analysis of a comprehensive network of more than 11,000 GNSS stations^37^, tide gauges and satellite altimetry over the period of 1995–2020 (Methods 6.1.4, Extended Data Figs. 1–3 and Extended Data Table 1). We estimate linear trends, a set of common modes of variability and station-dependent noise using a Bayesian principal component analysis (BPCA). The temporal variations of the common modes of variability are modelled using auto-correlated Gaussian Random Walks (GRWs; Extended Data Figs. 3 and 5). The continuous 3D (in space and time) VLM reconstruction is obtained from the sum of the re-combined GRWs and interpolated spatial weighting pattern and the secular trend estimates. Accordingly, the uncertainties of the individual components (trends and modes of variability) are propagated into the final reconstruction.

**Extended Data Fig. 4:**
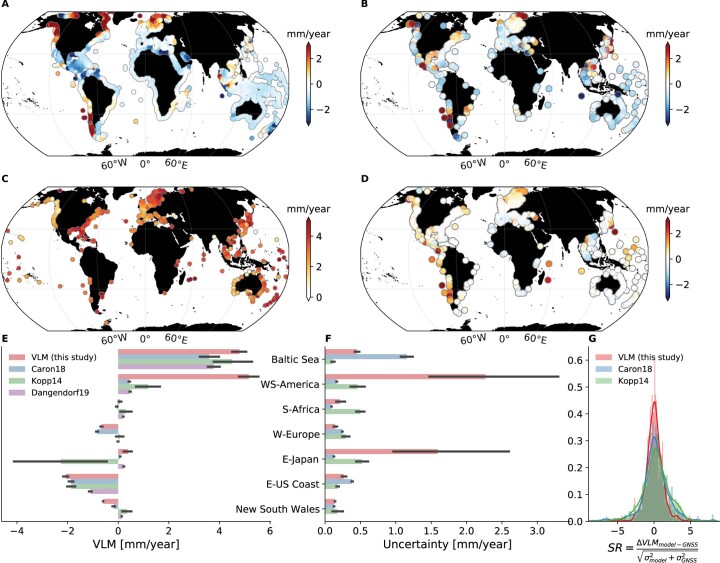
Validation of vertical land motion estimates. A and B show trend differences of vertical land motion (VLM) of this study and the estimates of recent sea level (SL) reconstructions^13,19^. Note, that the estimate of^13^ complies here with the glacial isostatic adjustment (GIA) fingerprint of their SL reconstruction, which is hence consistent with the estimate derived by ref. ^12^. (C) Absolute SL trends from altimetry over 1995–2020. D shows differences in the estimated uncertainties of the VLM reconstruction and those applied at tide gauges in the IPCC AR6 report^1,19^. E and F compare trend and uncertainty estimates from different solutions. Average contributions and 95% confidence intervals (representing variation within different regions) are shown. G shows the significance ratio of the trend differences $SR=\Delta VLM(model-$ $GNSS)/\sqrt{{\sigma }^{2}model+{\sigma }^{2}GNSS}$ of different model estimates w.r.t. to 775 GNSS trends and uncertainties estimated with MIDAS^60^. The standard deviation of SR are 1.2, 2.1 and 2.2 for the VLM reconstruction^13,19^.

This analysis allows us not only to disentangle the individual components (that is, trends and common modes of variability) but also to rigorously quantify uncertainties, associated with each of the components, and space/time-dependent uncertainties (BPCA; Methods). This approach advances state-of-the-art VLM estimates (for example, as currently used for sea-level (SL) projections), which lack any information of time-dependent VLM, and increases the number of observations by tenfold. As a result, based on the significance ratio, the trends and uncertainty estimates of our VLM reconstruction are about two times more accurate than the data currently employed in SL projections^38^ (validation in Methods and in Extended Data Fig. 4).

**Fig. 2:**
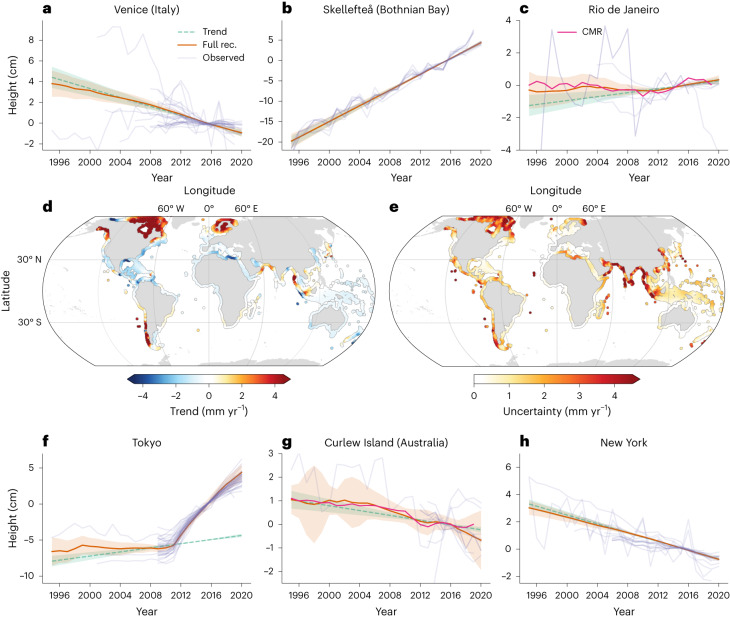
Bayesian VLM reconstruction from 1995 to 2020 based on observations. **a**–**c**,**f**–**h**, Time series of observed VLM at Venice (Italy) (**a**), Skellefteå (Bothnian Bay) (**b**), Rio de Janeiro (**c**), Tokyo (**f**), Curlew Island (Australia) (**g**), New York (**h**), together with the full VLM reconstruction (rec., red), 1*σ* uncertainties and the estimated trend component (green). Translucent purple time series indicate observed VLM in close vicinity (0.5–2° distances) to the depicted location to illustrate the spatio-temporal variability in the observations. The effect of CMR^22^ on VLM is shown by the pink line (**c**,**g**). **d**,**e**, Bayesian estimates of interpolated linear trends (**d**) and 1*σ* uncertainties (**e**).

The reconstructed coastal VLM (red lines) is compared to point-wise observations (purple lines) at selected sites in Fig. 2a–c,f-h. The estimated linear trends and uncertainties are represented by the green lines. When focusing only on the linear trends from reconstructed VLM (Fig. 2d,e), the spatial patterns largely reflect the GIA signatures, most visible in North America and Fennoscandia (Extended Data Fig. 6). These patterns are superimposed on regional non-GIA related effects, due to natural or anthropogenic causes. Using the reconstruction, we find subsidence along the coasts of the Gulf of Mexico (−1 to −7 mm yr^−1^) and the Australian coastlines (with average rates of −0.82 mm yr^−1^), which agrees with previous research^10,39,40^ and confirms systematic differences between observed subsidence rates and GIA model estimates.

We identify strong regional variations in the estimated trend uncertainties (Fig. 2e). In particular, in subduction zones (for example, in South America, Indonesia and Japan; Fig. 2e), increased trend uncertainties are associated with enhanced nonlinear variability in VLM. This finding is supported by Extended Data Fig. 7b, in which we show the standard deviations of the estimated nonlinear components of the VLM reconstruction, which are increased in these regions. Lower uncertainties (*<* 1 mm yr^−1^) are estimated for central Europe and the US East Coast, which is in accordance with a reduced estimated variance of nonlinear processes (Extended Data Fig. 7) and with higher station density.

Changes in coastal VLM are nonlinear at many locations, as evidenced in Fig. 2. At the coasts of Japan, earthquakes and the associated seismic deformation generate highly nonlinear VLM responses. One of the advantages of the BPCA is that the secular background trend (such as the inter-seismic trend) can be separated from the earthquake-related dynamics in contrast to previous global-scale analyses^2,4,21^. Knowledge of the secular background trends is essential to extrapolate VLM beyond the observational period in such a way that it is unbiased by present-day variability, in this case the motion during the postseismic deformation, which converges to the estimated inter-seismic velocity.

Also surface mass loading changes are attainable with the Bayesian VLM reconstruction. Predominantly hydrologically forced interannual VLM variations are visible in the VLM time series of Rio de Janeiro and Curlew Island, which correlate with the independently derived estimate of contemporary mass redistribution effects^22^ (with correlation coefficients of 0.5 and 0.9). The identification of these regional nonlinear processes is crucial to estimate the contribution of VLM to contemporary RSL change and its uncertainties.

## VLM contribution to contemporary regional sea-level change

**Fig. 3:**
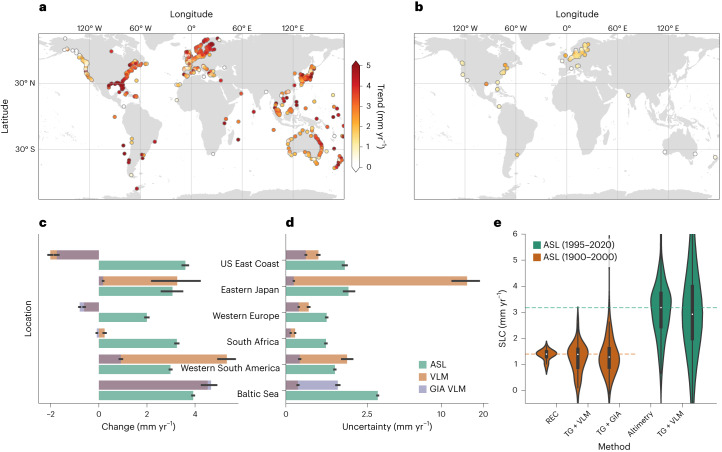
Contemporary regional sea-level changes. **a**, Absolute SL trends from vertical land motion (VLM)-adjusted tide gauges from 1995 to 2020. Here VLM is computed from the present-day VLM reconstruction, which includes the full effect of inferred interannual variability. **b**, Absolute SL trends computed over the last century (1900–2000), where tide gauges are adjusted for the linear trend component of the reconstruction excluding the present-day variability. **c**,**d**, The mean absolute SL and VLM trends (**c**) and uncertainties (**d**) for different macro regions (Methods provide for their spatial definition and number of samples), together with the averaged GIA model estimate along the coastlines for the period 1995–2020 (note the nonlinear scale for uncertainty components). Here the 95% confidence intervals of the point estimates within a region is indicated by the black lines. **e**, The distributions of contemporary absolute SL observations/estimates (from 1900 to 2000 and 1995 to 2020) for SL reconstructions, VLM-adjusted tide gauges (using both the VLM reconstruction and the GIA model estimate) and for altimetry observations (each for the same set of tide gauges). The inner boxplots shows the median, 25th and 75th percentiles, and the whiskers expand the quartiles by 1.5 times the interquartile range. The dashed lines indicate the median estimates of the SL reconstruction (brown) and the altimetry observations (green).

To understand the contribution of VLM to SL change over the twentieth century from regional to global scales, we adjust tide gauges for VLM and derive the absolute coastal sea-level change from 1995 to 2020 (Fig. 3a and Extended Data Fig. 4c) and 1900 to 2000 (Fig. 3b). We find high consistency between absolute SL trend from 1995–2020 inferred from 542 tide gauges and the regional coastal altimetry-based trends (which are interpolated onto the tide gauge location). Absolute SL trends averaged over all globally distributed tide gauges result in 3.09 mm yr^−1^ (median: 3.18 mm yr^−1^) using the closest altimetry point and 3.21 mm yr^−1^ (median: 2.93 mm yr^−1^) using VLM-adjusted tide gauge records for the period 1995–2020.

We quantify coastal averages of absolute SL trends, VLM and their uncertainties, which are computed from 1995 to 2020, at six different coastal macro regions (Fig. 3c,d). Note that the linear rates of VLM are computed from the full Bayesian reconstruction including the present-day VLM variability.

Subsidence in Western Europe and at the US East Coast adds to the absolute SL change and yields relative SL trends from 2.6 ± 1.0 mm yr^−1^ and 5.6 ± 1.6 mm yr^−1^, respectively, for the period 1995–2020 (Fig. 3c). A large share of the subsidence in western Europe and the US East Coast (between 31° N and 41° N) can be associated with the GIA forebulge collapse^4,41,42^. Compared with late Holocene geological rates^43^, both the GIA model and the VLM reconstruction predict stronger subsidence south of 40° N along the US East Coast (Extended Data Fig. 8). This was also reported in previous work^42^ and was attributed to the ongoing groundwater extraction in these areas. When taking into account its combined uncertainties, the inferred VLM does not significantly deviate from the GIA model^44^, averaged along the same coastal profiles (Extended Data Fig. 6). There is low temporal variability in these regions (Extended Data Fig. 7b), leading to similar uncertainty estimates as provided by the GIA model. Other regions are, however, subjected to tectonic processes that can nearly exponentially inflate uncertainties of VLM (note the nonlinear scale in Fig. 3d). This is particularly evident for eastern Japan and western South America, where tectonic uplift completely offsets the present absolute SL change.

Averaged along the global coastal profile shown in Fig. 2d, VLM explains a significant fraction of the variance of relative SL change (34%) and its uncertainties (26%) over the altimetry era. In total, the estimated uncertainties of the VLM reconstruction, which take into account nonlinear processes, are higher than the uncertainties provided by the GIA model^44^ (explaining 19% of the relative SL trend uncertainties).

The VLM reconstruction represents a crucial observation-based constraint to SL estimates over the last century. Hence, we explore the impact of VLM on relative SL change over the period 1900–2000 using 64 tide gauges with at least 80 years of data (Fig. 3b). Relative SL change at tide gauges is corrected for VLM in two ways: using the VLM reconstruction of this study and a GIA model. The effect of contemporary mass redistribution is subtracted from the ASL sea-level reconstruction and from both VLM datasets (Methods). We compare the outputs with absolute SL change from an independent global SL reconstruction^13^ for which we extract the same tide gauge locations.

**Extended Data Fig. 5:**
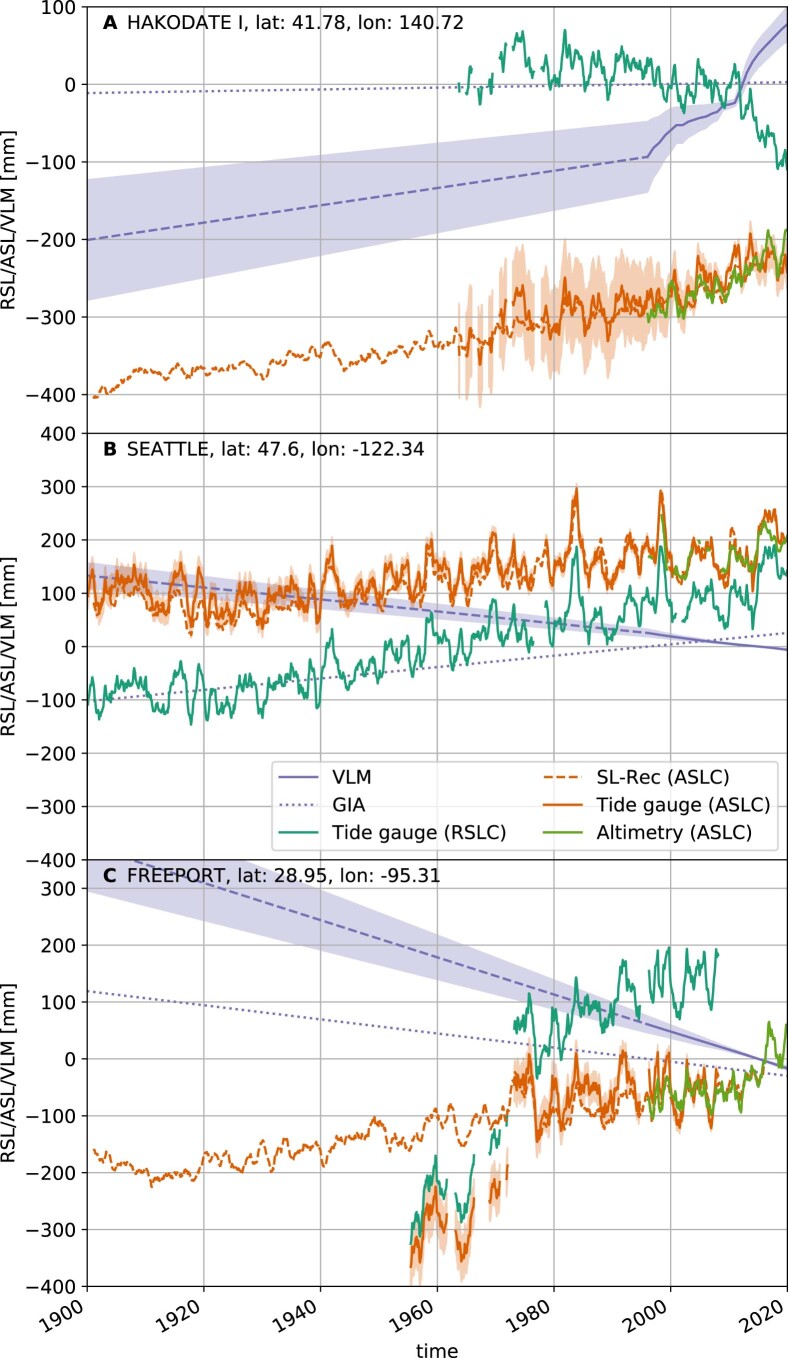
Sea level (SL) changes over the last century at tide gauges. Time series of relative SL change (dark green line) and vertical land motion (VLM)-adjusted absolute SL change from tide gauges (red solid line) in (**a**) Hokadate (Japan), (**b**) Seattle (USA), and (**c**) Freeport (USA). The present-day VLM estimate is shown by the blue solid line, the linear trend estimate (dashed line) represents the extrapolation of VLM back intime, the blue shaded area represents 1*σ* uncertainties, and the dotted line indicates the glacial isostatic adjustment model^44^ estimate at the tide gauge. The VLM uncertainties are added to the VLM-adjusted absolute SL change time series at tide gauges (red shadings). For comparison, the altimetry observations (green) and SL- reconstruction estimates^13^ (red dashed lines) over the last century are included.

The median absolute SL change estimates (from 1900 to 2000) for the SL reconstruction^13^ are 1.39 mm yr^−1^, 1.38 mm yr^−1^ for the VLM-adjusted tide gauges and 1.27 mm yr^−1^ for the GIA-adjusted tide gauges (Fig. 3e). Using bootstrapped confidence intervals, we find that none of these averages are significantly different from each other (at the 95% confidence level). This high agreement indicates that the linear rates of the VLM reconstruction derived in this study are suitable to be extrapolated back into the last century (at the location of the tide gauges), to constrain the VLM at tide gauges and to derive absolute sea-level estimates. It also supports the validity of indirectly inferring VLM rates from long tide gauge records in sea-level reconstructions. However, it should be noted, that these results are based on a relatively small subset of tide gauges, which are mostly located in northern Europe and the United States. These regions are found to be associated with relatively stable VLM in time, mostly due to GIA (after removing the effect of CMR), which facilitates the extrapolation of the rates back in time. Regions, which are affected by nonlinear VLM (such as the Gulf of Mexico, Australia or Japan) may be less suited to extrapolate VLM back in time, if VLM is derived only from a limited observational period (1995–2020) (Methods and Extended Data Fig. 5). Thus, there is an urgent need to investigate the extent to which nonlinear VLM effects are also present in century-long tide gauge (TG) records.

## Nonlinear VLM limits regional coastal sea-level projections

To quantify the different contributions of absolute SL change and VLM to projected coastal relative SL, we utilize the outputs of the Coupled Model Intercomparison Project Phase 6^45^, under the Shared Socioeconomic Pathway SSP2–4.5 (ref. ^46^). We consider here the ensemble median and 1*σ* confidence intervals of the integrated contributions from ice sheets, glaciers, land water storage and ocean dynamics^47,48^. We combine the projected absolute coastal SL with the long-term linear trends of the VLM reconstruction. To project the VLM uncertainties, we generate a 1,000-member ensemble of possible future trajectories modelled by GRWs starting from 2020 until 2150, which are informed by the parameters estimated to determine the present-day variability. To derive the final VLM uncertainties, we compute the root sum square of the standard deviation of the ensemble spread and the trend uncertainties estimated in the Bayesian model.

**Fig. 4:**
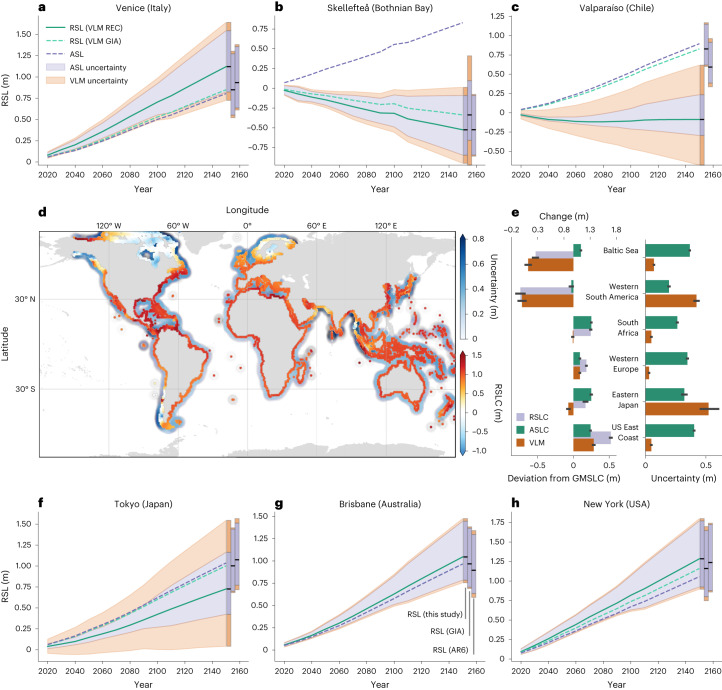
Overview of global and regional projected relative SL change. Overview based on IPCC AR6 projections^1,47,48^ (SSP2–4.5 scenario^46^), the VLM reconstruction and GIA model estimates^44^. **a**–**c**,**f**–**h**, Time series of the evolution of the estimated relative SL change at Venice (Italy) (**a**), Skellefteå (Bothnian Bay) (**b**), Valparaíso (Chile) (**c**), Tokyo (**f**), Brisbane (Australia) (**g**), New York (**h**), either based on the VLM reconstruction (solid teal line) or GIA model^44^ (dashed teal line) from 2020 to 2150. The absolute projected SL change is depicted by the blue dashed line and consists of the contributions of ice sheets, glaciers, land water storage and ocean dynamics^47,48^. Uncertainty contributions of the 1*σ* absolute SL change uncertainties and VLM (blue and orange shading) add up to the combined relative SL change uncertainties. The VLM uncertainties correspond to the square root of the sum of the squares of the standard deviation of the ensemble spread (of the Gaussian Random Walks) and the trend uncertainties estimated in the Bayesian model. **d**, The coastal projected relative SL change and the combined uncertainties (blue shading) of the absolute SL projections and the VLM reconstruction. **e**, Separates the different contributors of relative SL change and uncertainties for different regions. The relative SL change and absolute SL change are given in absolute terms and the deviation from the global mean sea-level rise in 2150. Average contributions and 95% confidence intervals (representing variation within different regions) are shown.

Figure 4a–c,f–h provide an overview of different projected local relative SL change estimates based on both the VLM reconstruction and GIA model at six different locations. GIA-induced uplift and subsidence strongly contribute to future SL in the Bothnian Bay (Skellefte) and the eastern US coast (New York), which is consistent with our VLM reconstruction. As shown in the previous analyses, unresolved processes in GIA models inevitably contribute to regional deviations of relative SL change projections in areas affected by localized subsidence (for example, in Venice, the Gulf of Mexico or the Nile Delta) or in regions impacted by high tectonic activity (western South America and Japan).

In Fig. 4e, we illustrate the regional future relative SL change in absolute terms and as the deviation from the global mean SL rise (0.8 m in 2150), obtained as the median of the ensemble model outputs. The bars depicting the VLM component indicate its estimated contribution to relative SL change. With respect to the altimetry era, the absolute explained spatial variance of coastal relative SL change by VLM is projected to decrease to about 22% in 2150 along the world’s coastlines, where coastal SL change is by implication more strongly dominated by the increased mean absolute SL rise. However, on a regional scale, VLM plays a predominant role in future SL change as shown in Fig. 4e. We estimate that VLM will explain 51% of the spatial variance of the relative SL deviations from the global mean in 2150. The regional impact of VLM is thus of the same magnitude as the combined responses to ocean dynamics and mass change fingerprints, causing a much larger range in the projected RSL rates (from −1 m to 1.5 m change in 2150), than the ASL change alone (0 m to 1.1 m; Extended Data Fig. 9). The bulk of the contribution of VLM is attributable to GIA^44^, which accounts for 41%. The influence of VLM remains of comparable importance when considering other radiative forcing scenarios, explaining 49% (for SSP3–7.0) and 47% (for SSP5–8.5) of the spatial variances of projected relative SL changes.

We find that VLM uncertainties explain a considerable proportion of relative SL change uncertainties (33%), corresponding to the combined uncertainties of the VLM and the 17th to 83rd percentiles of the ASL projections. Particularly large uncertainties in relative SL change projections, with values up to 1 m, are introduced in areas where nonlinear VLM is dominant, as exemplified in Extended Data Fig. 9b, or in Fig. 4c. Our uncertainty estimates in tectonically active regions (South America, Alaska, Japan) are systematically higher (by a factor of 2–5) than the coastal VLM uncertainties estimates, which are provided at tide gauges by the IPCC AR6 report^1,19^ (Extended Data Fig. 4d).

We provide evidence that the reconstructed VLM and uncertainty estimates more realistically reflect direct observations than alternative estimates^19,44^ because we explicitly take nonlinear effects into account (Extended Data Fig. 4h). By comparing different independent estimates of coastal VLM, we find that large discrepancies between these independent estimates (>10 mm yr^−1^) are associated with high temporal variability of VLM, as derived from the VLM reconstruction (Extended Data Fig. 7a). These regional errors due to large VLM variability must be compensated by large uncertainty ranges in RSL projections, which is ensured by our approach.

## The way forward

In this study, we incorporate observational VLM constraints to complete our picture of the spatio-temporal patterns of coastal VLM. Neglecting the effect of VLM in SL projections could lead to an underestimation of RSL in 2150 by up to 50 cm on a regional scale. Nonlinear VLM significantly raises the uncertainty limits of projected coastal SL change above previously reported levels^19^. We note that the underlying assumption here is that all contributing uncertainties (from VLM, ice sheets, thermal expansion and so on) are captured with equal accuracy. Currently this cannot be ensured, because of the different methodologies applied, which also differ for the individual ASL components (emulators, process models, Atmosphere-Ocean General Circulation Model ensembles and so on; refs. ^1,15^) and because of the structural uncertainty in ASL projections^47^. The choice is therefore to follow current practice in the determination of these uncertainties.

Another caveat is that in this work we project the linear VLM rates and estimate uncertainties based on the information of present-day variability. However, our results reinforce that it is not clear that VLM will continue to be linear everywhere and that the observed variability of VLM is a robust predictor of future behaviour. Some processes, such as earthquakes, which can cause nearly instantaneous vertical displacements on the order of metres, or changes in human-induced subsurface fluid withdrawal rates, make it difficult or even impossible to generate meaningful future predictions^49,50^. It is therefore possible that current SL projections^1^, including those provided in this work, still underestimate the uncertainty introduced by unpredictable VLM processes. Another remaining challenge is the low station coverage, in particular in highly populated regions, which are especially vulnerable due to high subsidence rates^36,51^. Therefore, recent studies have increasingly expanded VLM observations in space using InSAR to enable local SL projections at an unprecedented spatial resolution^30,51,52^. However, because VLM from InSAR is still relatively imprecise, covers only short periods of time and requires local geodetic ties, large networks of extended and high-quality VLM observations are indispensable to enhancing our understanding of the mechanisms shaping regional and nonlinear VLM. Our data-driven reconstruction is a crucial step in that direction.

## Methods

### Datasets

To reconstruct VLM, we combine GNSS VLM time series and indirect VLM estimates inferred from the differences of relative sea-level observations from tide gauges and absolute sea-level measurements from altimetry (hereinafter called SATTG), an approach that was steadily developed and extensively used over the last decades (for example, refs. ^2,53–57^). Although GNSS VLM data provide more accurate VLM information^58^ compared with SATTG^56,57^, SATTG time series are essential to increase the station density in coastal regions and to constrain the VLM reconstruction back in time. Whereas SATTG time series can be computed from approximately 1992 (at the start of the TOPEX/Poseidon mission), most (∼ 90%) of the available GNSS time series start after the year 2002^4^. As a compromise, considering the limitations in data availability before 2000, the VLM reconstruction is computed over 1995–2020.

#### GNSS data

We use 24-hour GNSS position time series from the Nevada Geodetic Laboratory (NGL) of the University of Nevada (http://geodesy.unr.edu)^37^ aligned to the IGS14 reference frame^59^. We select time series with a duration of at least five years and two years of valid data. We omit time series with an absolute trend larger than 20 mm yr^−1^ and a trend uncertainty higher than 3 mm yr^−1^ (based on MIDAS (Median Interannual Difference Adjusted for Skewness)^60^ trend and uncertainty estimates), similarly as done in previous studies (for example, ref. ^20^). With this selection we aim to reject time series, which are probably associated with very localized extreme VLM, which are not representative for regional-scale VLM and might otherwise increase uncertainties in the interpolated maps (due to higher spatial variance of the trends). As an example, less than 2% of the trends (estimated with MIDAS) have magnitude greater 20 mm yr^−1^ and are randomly distributed over the globe, which supports that these cases represent individual very local movements. After applying the subsequently described post-processing, we obtain a global dataset consisting of 10,957 station time series.

#### Tide gauge data

We use 713 monthly tide gauge time series from the revised local reference PSMSL database^61^. The revised local reference data present the commonly used data in sea-level analyses, as they are quality checked for errors such as datum inconsistencies, or jumps, and are additionally compared with respect to records of neighbouring tide gauge stations^61^. The tide gauges are corrected for dynamic atmospheric correction^62^ and tides (FES2014^63^). Time series with less than 120 months of valid data (over 1995–2020) are rejected from the analysis.

#### SATTG data

SATTG VLM time series are computed based on monthly PSMSL tide gauges^61^ and gridded 0.25 satellite altimetry data (SAT) (https://resources.marine.copernicus.eu/product-detail/SEALEVEL_GLO_PHY_L4_MY_008_047/INFORMATION). The monthly altimetry SL anomalies are available from 1 January 1993 to 31 December 2020. The combination of SATTG data is optimized by using the highest correlated grid point with respect to monthly tide gauge observations (based on de-trended and de-seasoned data) (for example, refs. ^2,54,58,64^) because it decreases the discrepancies between the observed oceanic signal of altimetry and tide gauges and thus the noise in the time series. It should be noted that the noise level in the resulting SATTG time series is still a magnitude larger than in GNSS data, which can also be further inflated by instrumental issues of both systems (SAT and tide gauges). We only keep SATTG time series with at least 120 months of valid data.

#### Sea-level reconstructions

To investigate implications of the VLM reconstruction for contemporary SL change estimates, we compare VLM corrected tide gauge data with the SL reconstruction by ref. ^13^. The sea-level reconstruction combines several observations (tide gauges, satellite altimetry) and model estimates (for example, from GIA models and global climate models) to resolve the spatio-temporal global sea-level variations during 1900–2015. Here relative sea-level change data from tide gauge observations are used as constraints to estimate the superimposed contributions (from short- and long-term sea-level changes and VLM) to global sea-level changes. The hybrid reconstruction combines low-frequency SL information based on the Kalman Smoother (KS)^12^ and high-frequency SL estimates derived with the Reduced Space Optimal Interpolation technique^11,12^. The KS approach fits known SL fingerprints (present-day ice melting, GIA and dynamic sea surface height pattern) to a set of 622 long tide gauge records and provides estimates of the century-long SL change. In the KS, a term addressing the local non-climatic factors (such as VLM) with a decorrelation length scale of 1,500 km was considered^12^. We note, however, that the fields used in the hybrid reconstruction^13^ were solely based on the weighted sum of ice melt and ocean dynamics. Thus they are not directly affected by any smearing effects from the residual term^65^.

#### Sea-level projections

We use sea-level projections of the IPCC AR6^1^ report, based on climate model results of the Coupled Model Intercomparison Project Phase 6 (https://esgf-node.llnl.gov/search/cmip6/)^45^. We explore different scenarios (SSP2–4.5, SSP3–7.0 and SSP5–8.5^46^), each relying on different emission scenarios. We particularly focus on the SSP2–4.5 scenario, which represents the medium pathway of future emissions in which CO_2_ levels are assumed to decline mid-century, causing a temperature increase of 2.7° in 2100. Higher greenhouse gas emissions are assumed for SSP3–7.0 and SSP5–8.5 scenarios, which are associated with a radiative forcing of 7 W m^−2^ and 8.5 W m^−2^ or global CO_2_ concentrations of more than 800 or 1,100 ppm by the year 2100^46^. We consider the ensemble median and one-sigma credible intervals (based on the 17th to 83rd percentiles) of the integrated contributions from ice sheets, glaciers, land water storage and ocean dynamics^47,48^. The outputs are provided at tide gauge locations and as maps with a 1-degree spatial resolution with ten-year temporal sampling.

#### GIA vertical land motion

We incorporate a recent GIA trend and formal uncertainty estimate by ref. ^44^, which was derived from Bayesian inferences of the probability distributions of model parameters describing the rheological structure of the Earth and ice history. The solution was constrained by 11,451 relative sea-level change records and 459 GNSS-based trends. The model is based on solving the classical loading equations for a compressible Maxwell solid Earth and surface loading due to ice sheet mass changes during the last 122 kyr (ref. ^44^) used spherical harmonic truncated at degree 89 (1° spatial resolution) in the centre of mass frame and included feedbacks from Earth rotation and the resolution of the sea-level equation. To generate a likelihood probability distribution of the model parameters, they generated an ensemble of 127,000 forward models (with different parameters) and estimated the probability of how likely a given model was to explain the observational constraints. The VLM estimates correspond to the expectation and standard deviation of these probability distributions. These GIA fields are combined with contemporary sea-level changes and also with sea-level projections.

#### Vertical land motion caused by contemporary mass redistribution

Mass changes in glaciers and ice sheets or land water storage changes cause elastic responses of the Earth, generating partially nonlinear VLM (for example, refs. ^6,66^). We use a gridded annual estimate (from 1900 to 2018) of the effect of contemporary surface mass redistribution (CMR) on VLM as provided by ref. ^22^. From 2003 to 2018, the estimate is based on a combination of GRACE (Gravity Recovery and Climate Experiment^67^) and GRACE-FO (Gravity Recovery and Climate Experiment Follow-On^68^). Before 2003, the solution relies on process model estimates of the mass changes of glaciers, ice sheets and terrestrial water storage. We compare the CMR estimates with the VLM reconstruction from 1995 to 2020 (Extended Data Fig. 2) and apply the CMR correction to century-long tide gauge records (corrected for the VLM reconstruction and GIA) as shown in Extended Data Fig. 3e.

### Reconstruction of time- and space-variable vertical land motion

To understand the impact of VLM on contemporary and projected relative SL change, we resolve VLM continuously in space and time. We apply a three-step procedure to derive the spatially and temporally varying VLM reconstruction. First, the input data (GNSS, SATTG) is preprocessed, meaning that the time series are adjusted/corrected for offsets, single point outliers and the annual cycle in a semi-automated manner. Second, we perform a dimensionality reduction of the spatio-temporal variability of the data by estimating long-term linear trends and common modes of variability using a Bayesian principal component analysis (BPCA^69^). Finally, we spatially interpolate the approximated linear trends and Empirical Orthogonal Functions (EOFs) and their associated uncertainties using an adaptive Bayesian transdimensional regression approach^21^. The continuous 3D (in space and time) VLM reconstruction is obtained from sum of the re-combined principal components (PCs) and interpolated EOF pattern and the secular trend estimates. Accordingly, as the uncertainty maps of the trend and EOF pattern are individually derived, we apply an uncertainty propagation of the different components to derive the space/time-dependent uncertainties. Extended Data Fig. 1 illustrates the corresponding processing chain of the data. Details of each step are provided in the following.

#### Pre-processing vertical land motion data

Several pre-processing steps have to be applied before physically meaningful modes of variability can be derived from the combined GNSS and SATTG data. To reconstruct smoothly varying elevation changes, we remove discontinuities from the data. In particular, GNSS data can be affected by discontinuities due to instrumental issues^70^. Discontinuities are, however, less frequent and harder to detect in SATTG time series, due to the higher noise amplitudes in the data. To support the identification of discontinuities, we apply DiscoTimeS (discontinuities in time series^14^) as an unsupervised discontinuity detector. DiscoTimeS identifies discontinuities and trend changes and the annual cycle and noise properties. The simultaneous estimation of discontinuities and trend changes is particularly important in regions where decadal rate variations are evident. DiscoTimeS is applied to weekly downsampled GNSS time series and monthly SATTG data.

We manually revise the unsupervised time series fits and reject estimated discontinuities in the case of misfitting or oversegmentation of the data. We also reject stations after visual screening of the time series (as is common practice; for example, refs. ^2,71^) and apply an outlier analysis based on the regional variability of trends. Any remaining outliers, for example, caused by local effects, such as settling of the installation platform or building, are further reduced with the spatial interpolation of neighbouring data. Problematic stations that are located on ice, for instance, are excluded according to the selection by ref. ^20^. The Data Appendix provides a complete list of station IDs used.

The time series are subsequently corrected for discontinuities and the annual cycle. Remaining single point outliers (in the time series) are rejected by removing values which exceed a running mean standard deviation outlier test. For this purpose, we first compute the median of the 12-month running standard deviation (*σ*_12m_), which provides a measure of variability that is not influenced by potential remaining outliers in the data. Next, we reject values whose difference with respect to the 12-month running mean is three times larger than *σ*_12m_. Finally, because we focus on interannual to decadal VLM variations, we compute annual averages to obtain a homogeneous sampling. We obtain 11,670 time series (10,957 GNSS and 713 SATTG time series) with at least five years of valid data (Fig. 2). To align the station-dependent absolute height differences, we compute height changes with respect to the value in 2014.

#### Bayesian principal component analysis

The principle of our approach is to capture common modes of spatio-temporal variability of VLM on interannual to decadal timescales, together with underlying long-term trends. The main drivers of interannual/decadal variability are tectonic activity, surface mass loading changes (for example, from terrestrial water storage changes) or local effects such as groundwater depletion. Previous studies^5,6^ demonstrated that these processes exhibit spatially coherent variations in the order of several 100 km. Thus, to disentangle and describe these modes, we utilize a principal component analysis (PCA).

PCA analysis was previously applied to GNSS data to identify a Common Mode Error to reduce its associated effect on networks of GNSS data^40,70,71^. To overcome the problem of missing values in time series, previous studies employed probabilistic PCA (PPCA), which allows approximating the principal components for discontinuous data^71^. Here we utilize PPCA, more precisely Bayesian PCA (BPCA), as it has the advantage of estimating a full posterior probability distribution of the parameters^69^, in contrast to maximum likelihood estimation^71^.

We estimate heights *U*(*x*,*t*) at every station location **x** and time **t** as described by the following process model:

1 $$U(x,t)=N\left({g}_{x}t+\mathop{\sum }\limits_{k=1}^{n}{W}_{k,x}{p}_{k,t},{{\epsilon }}_{x}^{2}\right)$$

Here ${{x}}=({x}_{1},{x}_{2},\ldots ,{x}_{s})$^*T*^ denotes a set of *s* stations (GNSS or TG stations), whereby *s* varies across the considered regions, as explained below. Vector **t** contains 26 years from 1995 to 2020. *p*_*k*,*t*_ are latent variables, or principal components, which are mapped onto the observations by the transformation matrix ${W}_{k,x}.$ ${W}_{k,x}$ represents the spatial pattern of the common modes of variability (that is, the EOF pattern), whereas the principal components *p*_*k*,*t*_ modulate the evolution of these pattern over time. The vector **g** accounts for constant linear trends in the time series. The technique-dependent variance ${\epsilon }_{x}^{\;{2}}$ is estimated individually for the two different techniques (GNSS and SATTG), considering that noise amplitudes differ by one order of magnitude.

For each parameter, we define prior distributions. We assign Gaussian distributions to *g*_*x*_ and *W*_*k*,*x*_ and a half-normal distribution for the estimated variance ${\epsilon }_{x}$:

2 $$P({\bf{g}})=N({\mu }_{g},{\sigma }_{g}^{2})$$

3 $$P({\bf{W}}\,)=N({\,\mu }_{W},{\sigma }_{W}^{2})$$

4 $$P({\mathbf{\upepsilon}})=\mathrm{half}N({\sigma }_{\epsilon }^{2})$$

The principal components are modelled as Gaussian Random Walks to simulate smoothly varying behaviour of the VLM. In doing so, the principal components represent auto-correlated time series. With this constraint, we avoid that spurious high-frequency signals are absorbed by the PCs. Note that discontinuities are removed from the data before applying the BPCA, which otherwise would lead to an overestimation of the variance of the principal components. In our case, the principal component *p*_*k*_ at time step *t* is obtained by adding the random normally distributed innovation *h*_*k*_ at time step *t* − 1, as summarized by the following formula:

5 $${p}_{k,t}={p}_{k,t-1}+{h}_{k,t},P\left({h}_{k}\right)=N\left({\mu }_{{h}_{k}},{\sigma }_{{h}_{k}}^{2}\right)$$

Different prior assumptions are assigned to the unknown parameters **Φ**_*b*_. We initialize the point-wise trends *μ*_*g*_ with linear trend estimates derived with ordinary least squares analysis and set ${\sigma }_{g}^{\,{2}}$ to 10 mm yr^−1^. *μ*_*W*_ is set to zero, and *σ*_*W*_^2^ is set to 15 cm. ${\sigma }_{{h}_{k}}^{2}$ is set to 0.001 for the first component (*k* = 1) and ${\sigma }_{{h}_{k}}^{2}=\frac{{\sigma }_{{h}_{k-1}}^{2}}{2}$ for every subsequent component, such that the prescribed amplitudes of the Gaussian Random Walks decay with increasing number of components. Finally, we assign ${\sigma }_{\epsilon }^{\,{2}}$ to 40 cm.

We perform the BPCA for several continental subregions. These include North America (*s* = 5,414), Europe (*s* = 3,242), Oceania (Australia, New Zealand and Southeast Asia, *s* = 980), Japan (*s* = 1,212), Africa (*s* = 272), South/Central Asia (*s* = 153) and South America (*s* = 550). We applied this regional separation to maximize the explained temporal VLM variability depending on the given regional processes. Distinct differences between the modes of variability are particularly caused by tectonic activity. We determine the maximum number of used PCs *n* by repeatedly simulating different numbers of PCs. If the weighted explained variance EV of the data **U**_obs_ by the model **U**_model_ does not improve significantly (that is, when the improvement is below 1–2%) by adding new PCs, we stop the iteration. The explained variance is computed by taking into account weights **w** associated with the regional distribution of the data (that is, the station density **d**) and the technique-dependent estimated variance parameter (equation (6)). This avoids an over-representation of variability in regions with high station coverage or very noisy SATTG data.

6 $$\mathrm{EV}=1-\frac{\sum\mathrm{Var}({{\bf{U}}}_\mathrm{obs}-{{\bf{U}}}_\mathrm{model}){\bf{w}}}{\sum\mathrm{Var}({{\bf{U}}}_\mathrm{obs}){\bf{w}}},{\bf{w}}=\frac{1}{{\bf{\upepsilon}}{\bf{d}}}$$

We use the state-of-the-art No-U-Turn (NUTS) sampler to generate inferences about the desired target distribution^72^. To test whether the chains have converged, we consider here the potential scale reduction factor $\widehat{R}$ and the relative effective sample size ESS per iteration *n*. $\widehat{R}$ is a widely used diagnostic that provides a measure of the standard deviation across chains, versus the within-chain variability. If $\widehat{R}$ does not converge to one, it indicates that the chains are unlikely to have converged to the equilibrium distribution^73,74^. ESS is a measure of the number independent samples within the chain, which is influenced by the auto-correlation of the samples. The averaged (in time or space) model diagnostic $\widehat{R}$ is close to one for the parameters **W**, **p** and **g**, indicating good mixing of the chains. ESS/*n* is greater than 0.1, which ensures that there are enough independent samples in the Markov chains and that the Monte Carlo error is sufficiently small^75^. NUTS provides additional useful convergence diagnostics which can indicate that the sampling from the posterior distribution is biased (divergent transitions) or inefficient (tree depth). There are no divergent transitions in the individual chains (for the different regions), and the maximum tree depth used (15) was not saturated.

#### Bayesian transdimensional regression and re-combination

The BPCA method yields a reconstruction of the evolution of VLM in time at every point-wise station. To obtain continuous estimates of VLM in space, we interpolate the estimated trend and EOF pattern using a Bayesian transdimensional approach^20,76,77^ as developed by ref. ^21^. A major advantage of this Bayesian framework is that an explicit regularization of the model parameters, in particular the definition of the spatial resolution, is not required and it is performed automatically by the algorithm^78^. This is advantageous compared with interpolation methods, which rely on a fixed number of model parameters or user-defined interpolation length scales.

As in ref. ^21^, we apply a Delaunay linear interpolation to recover smooth surfaces of the parameters (*W*_*k*,*x*_ and *g*_*x*_). We use the posterior averages of the estimated parameters and standard deviations *σ*_*b*,*x*_ of the individual spatial parameters (*W*_*k*,*x*_ and *g*_*x*_) of the BPCA as input parameters of the Bayesian regression. The Bayesian regression estimates the parameter values and statistical uncertainties, while dynamically adapting the complexity, that is, the spatial resolution of the grid or the number of mobile nodes, which depends on the spatial distribution (density of the data). The posterior probability distributions of the unknown parameters (trend and EOF surfaces) are approximated using a hybrid of Markov chain Monte Carlo and Hamilton Monte Carlo techniques^21,79,80^. Thus, the interpolated parameters (and their uncertainties) are directly estimated from the model ensemble (and spread), which consists of many different grid realizations. The Gaussian probability function of the unknown spatially interpolated parameters **Φ** given the point-wise input data, that is, the posterior means of the parameters $\bar{{{\boldsymbol{\Phi }}}_{b}}$ and their uncertainties *σ*_*b*,*x*_ is:

7 $$P({\boldsymbol{\Phi }}|\overline{{{\boldsymbol{\Phi }}}_{b}})=\frac{1}{{\sum }_{x}\sqrt{2\pi \lambda {\sigma }_{b,x}}}\exp\left\{-\sum _{x}\frac{{(\;f{(\Phi )}_{x}-\overline{{{\Phi }}_{b,x}})}^{2}}{2{\sigma }_{b,x}^{\,{2}}}\right\}$$

Here, *f*(Φ) is the forward model, that is, the Delaunay parameterization with a linear interpolant^21^ and *σ*_b,x_ is the estimated error derived in the BPCA analysis. *λ* is a hierarchical error scaling factor, which accounts for under or overestimation of the parameter errors, to account for small theoretical errors not sufficiently captured by *σ*_b,x_ (refs. ^77^ or ^21^).

We run 56 independent Markov chains. We start from randomized initial conditions, drawn from the prior distribution of the parameters. We use a uniform prior of VLM rates between ±15 mm yr^−1^. Every chain is run for 1,000,000 iterations where only the last 500,000 iterations are retained and averaged. At every iteration of the Markov chain, the model state is perturbed, which involves the variation of the number and distribution of the grid nodes. Thus, every Markov chain consists of a large ensemble of model states, which form the basis to compute the full posterior distribution. From this distribution, parameter uncertainties are derived. On the basis of the obtained interpolated 0.25° 2D maps, we retain regularly spaced coastal profiles of 0.25° resolution. We calculate the uncertainty propagation by incorporating the estimated errors of the interpolated trend and EOF pattern and the time-dependent errors of the PCs.

Extended Data Fig. 3 gives an overview of the different intermediate results obtained after the BPCA and the 2D interpolation in the region of Japan prone to seismic activity. Illustrated are the estimated PCs (Extended Data Fig. 3), together with the point-wise and interpolated EOFs and trends and their uncertainties (Extended Data Fig. 3). The spatial uncertainty estimates are a function of the station density, the spatial scatter of the data and their formal uncertainties. As an example, lower station density particularly contributes to increased uncertainties of the EOF pattern in the Tohoku region.

#### Validation

The point-wise BPCA VLM estimates capture most of the variance with regional values ranging from 87.1% to 98.5% of the observational database (GNSS and SATTG time series), as shown in Extended Data Table 1.

We compare coastal linear trend estimates of the interpolated VLM reconstruction with GIA estimates^44^ and VLM inferences from the SL reconstruction^19^, which were applied in the AR6 IPCC report^1^. The different VLM estimates are compared with GNSS trends^60^ at 775 tide gauges in Extended Data Fig. 4. Note that these GNSS trends were computed with MIDAS^60^, which accounts for discontinuities but assumes a constant linear trend over the period of observation. Therefore, these trends can potentially be influenced by nonlinear VLM, which are not represented by the GIA model, for instance. We computed the significance ratio of the trend differences with respect to the combined uncertainties $\mathrm{SR}=\Delta\mathrm{VLM}\,(\mathrm{model}-\mathrm{GNSS})/\sqrt{{\sigma }^{2}\mathrm{model}+{\sigma }^{2}\mathrm{GNSS}}$. A significance ratio of SR < 1 indicates that the trend differences are within the estimated limits of uncertainties and thus they are not significant. This ratio is a useful statistic to evaluate the accuracy of both the estimated trends and uncertainties. Extended Data Fig. 4g displays the distributions of SR for the different datasets. The standard deviations of SR are 1.2, 2.1 and 2.2 for the VLM reconstruction of this study, for ref. ^19^, and for ref. ^44^. Here a higher SR points to either higher trend differences or underestimated uncertainties in refs. ^19,44^. The standard deviations of the trend differences ΔVLM_model-GNSS_ (irrespective of the uncertainties) are 1.9, 2.5 and 2.5 mm yr^−1^ for the VLM reconstruction^19,44^. Therefore, the VLM reconstruction based on BPCA and Bayesian transdimensional regression computed in this study provides more realistic estimates of both VLM trends and uncertainties. Because a portion of the validation data is included in the VLM reconstruction, this comparison validates the statistical approach of the VLM reconstruction rather than the underlying database, which contains much more and higher quality VLM data than the other VLM datasets. Hence, with this analysis we aim to emphasize the discrepancies between contemporary VLM changes and previously applied estimates, which do not include any GNSS data and which do not account for nonlinear effects.

Regional differences between the VLM reconstruction and refs. ^13,19^ are shown in Fig. 4a,b. Here the VLM estimate of ref. ^13^ was derived from the difference of the provided absolute and relative SL trends and thus complies with the VLM fingerprint of GIA^12^. Note, that the absolute sea-level data in ref. ^13^ contain the deformational component of CMR. Extended Data Fig. 4d shows differences between the estimated long-term trend uncertainties of the VLM reconstruction and those provided by ref. ^19^. In addition, we provide a comparison of regional averages of VLM and uncertainty estimates in Fig. 4e,f. These arithmetic averages are computed based on the values on the coastal grid points for following regions: Baltic Sea (13–31.4° N, 52.7–66° E, *s* = 577), western South America (50–18.8° S, 87–68.8° W, *s* = 309), South Africa (37.5–27.2° S, 15.5–33.8° E, *s* = 104), western Europe (38–50° N, 15–0° E, *s* = 152), eastern Japan (24.5–40.2° N, 140.2148.6° E, *s* = 40), US East Coast (31.6–41.4° N, 82.3–68.2° W, *s* = 141), New South Wales (38.3–28.2° S, 149.8–154.4° E, *s* = 47). Here, *s* is the number of data points provided in the VLM reconstruction. Further validation experiments of our approach can be found in the supplementary file.

#### Comparison with century-long tide gauge time series

To exemplify how nonlinear and non-GIA VLM impacts the calculation of past SL change, we show the results in three different tide gauge time series in Extended Data Fig. 5. First, Extended Data Fig. 5a addresses the effect of nonlinear VLM (blue line) on relative SL change measured by the tide gauge (dark green line) subjected to tectonic activity. Here the combined effect of inferred VLM and relative SL change shows high agreement with the absolute SL observations from altimetry. To extrapolate VLM back in time, we use the estimated linear trend component (and associated uncertainties) from the BPCA analysis. The adjusted absolute SL (1965–1995) estimate of the tide gauge (Extended Data Fig. 5a) is consistent with independent absolute SL change estimates from a recent sea-level reconstruction^13^. This corroborates that for the considered tide gauge, the separation of the time-varying present-day VLM and a secular background trend provides valuable information on past SL changes.

**Extended Data Fig. 6:**
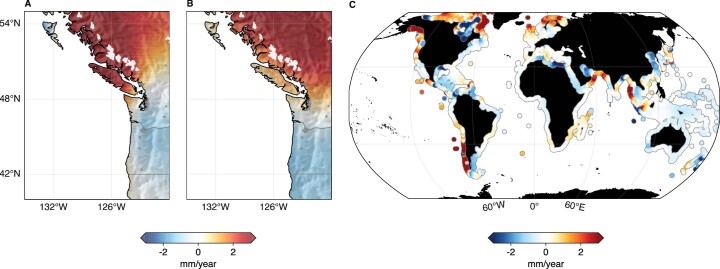
Comparison with glacial isostatic adjustment (GIA) model estimates. (**a**) VLM reconstruction, (**b**) GIA estimate^44^, (**c**) differences of the VLM reconstruction and the GIA estimate.

**Extended Data Fig. 7:**
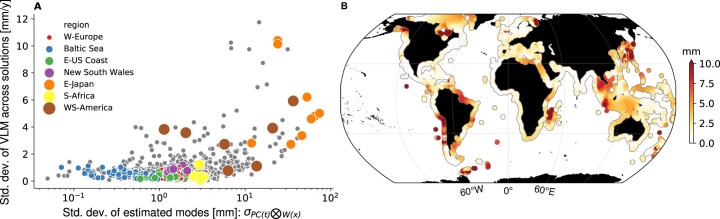
Present-day vertical land motion variability. (**a**) illustrates how time-variable VLM relates to differences in vertical land motion (VLM) estimates across different solutions. Here, the y-axis shows the standard deviations of different VLM estimates (that is, the VLM reconstruction^13,19,44^, and GNSS trends) computed at 755 different coastal GNSS stations. The x-axis (note the logarithmic scale) sorts the data according to the estimated standard deviation (in time) of the present-day VLM variability of the reconstruction ${\sigma }_{PC\otimes EOF}$ (that is, the variability estimated by the EOFs). Time-variable VLM increases the probability of discrepancies between different, VLM estimates. (**b**) depicts the estimated coastal present-day VLM variability ${\sigma }_{PC\otimes EOF}$.

**Extended Data Fig. 8:**
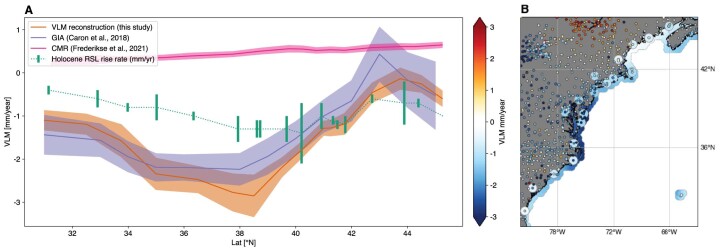
Comparison of vertical land motion estimates along the US East Coast. (**a**) comparison of VLM (and its uncertainty) from glacial isostatic adjustment (GIA^44^, the vertical land motion (VLM) reconstruction (orange), contemporary mass redistribution (CMR, pink^22^) with 1*σ* uncertainties, and late Holocene records from the last 4000 years (green^43^) as a function of latitude along the US East Coast (as in ref. ^42^). The green bars denote the 2*σ* uncertainties of the Holocene rates. (**b**) indicates the GNSS and SATTG (altimetry minus tide gauge) rates used in this study as small circles, the geological rates as large circles^43^, as well as the coastal profile of the VLM reconstruction along the coastline.

**Extended Data Fig. 9:**
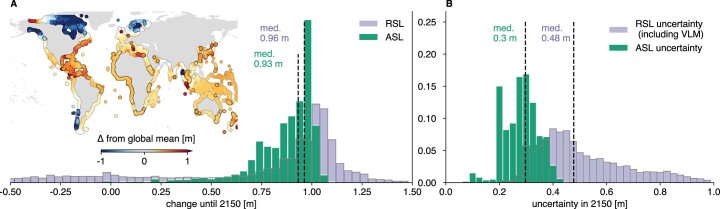
Components of projected sea-level change. **a**) Histogram of projected coastal absolute sea level (ASL, SSP2-4.5 scenario^1,48^) and relative sea level (RSL) changes until 2150 [m]. The map indicates deviations of RSL changes from the global coastal mean RSL change. The black circles highlight where these differences are significant (based on the combined 1*σ* uncertainties). The dashed lines display the median values of the distributions. (**b**) Same as in (A) but for the estimated RSL and ASL change uncertainties in 2150.

Second, Extended Data Fig. 5b is a notable example of VLM controlled by plate-tectonic processes and GIA^81,82^. The tide gauge in Seattle shows a subsidence of the order of 1 mm yr^−1^, which deviates strongly from the predicted uplift by the GIA model (Extended Data Fig. 5b and Extended Data Fig. 6). This deviation is mainly caused by the mismatch of the amplitude and the spatial structure of the North–South gradient of observed VLM and GIA model estimates (for example, ref. ^81^). The high agreement of the VLM-adjusted tide gauge observations and reconstructed sea level suggests constant VLM rates over the last century at this location. This is also supported by geological data, which indicate steady subsidence rates over the last 5,000 years^83,84^.

The third case displays the Freeport tide gauge located at the Gulf of Mexico (Extended Data Fig. 5c), which is affected by nonlinear VLM due to withdrawal of hydrocarbons and groundwater^10,85^. Whereas tide gauge relative SL change and observed VLM both display a constant pace after 1970, the relative SL change before that period indicates either substantially increased subsidence rates (on the order of cm per year) or even instantaneous vertical displacement of the tide gauge. Similar nonlinear rates were observed at the Galveston tide gauge^9^, which is relatively close (64 km) to Freeport. Thus, on regional to local scales, unobserved effects of nonlinear VLM (mainly due to tectonic processes and human activities) introduce large uncertainties in SL reconstructions and can significantly exceed the estimated Bayesian model 1*σ* uncertainty intervals (which are added to the re-estimated absolute SL in Extended Data Fig. 5c). This confirms the importance of identifying present-day VLM variability, which is paramount for aligning tide gauge and altimetry observations and to prevent biases in extrapolated observed VLM due to nonlinear effects.

## Online content

Any methods, additional references, Nature Portfolio reporting summaries, source data, extended data, supplementary information, acknowledgements, peer review information; details of author contributions and competing interests; and statements of data and code availability are available at 10.1038/s41561-023-01357-2.

## Supplementary information

Supplementary Information Supplementary Methods.

## Data Availability

The vertical land motion reconstruction (including the different trend components, modes of present-day variability and point-wise estimates) are available at Zenodo, 10.5281/zenodo.8308347. The NGL-GNSS data are obtained from http://geodesy.unr.edu (ref. ^61^). Monthly tide gauge data from PSMSL are available at https://www.psmsl.org/data/obtaining/ (ref. ^62^). Gridded altimetry data are available at https://resources.marine.copernicus.eu/product-detail/SEALEVEL_GLO_PHY_L4_MY_008_047/INFORMATION. The GIA dataset is available at JPL/NASA (https://vesl.jpl.nasa.gov/solid-earth/gia/ ref. ^44^). The contemporary mass redistribution data is available at Zenodo (10.5281/zenodo.3862995 ref. ^41^).

## Extended data

**Extended Data Table 1:** Explained weighted variance of estimated VLM and number of estimated PCs

Region	Expl. Variance [%]	Number of PCs used
North America	96.2	2
Europe	94.0	2
South America	89.6	2
Japan	98.5	3
Oceania	87.1	3
Africa	94.6	2
South/Central Asia	87.5	3

## Code availability

The codes for the VLM reconstruction are available on Github (https://github.com/oelsmann/discotimes and https://github.com/oelsmann/bpca). The code of the transdimensional regression is available at https://github.com/rhyshawkins/TransTessellate2D. We also used the Global Self-consistent, Hierarchical, High-resolution Geography Database (GSHHG)^86^ as third-party data to draw continental coastlines in Figs. 2–4, and Extended Data Figs. 2–4 and 6–9.

## References

[1] Fox-Kemper, B. et al. in *Climate Change 2021: The Physical Science Basis* (eds Masson-Delmotte, V. et al.) Ch. 9 (Cambridge Univ. Press, 2021).

[2] Wöppelmann, G. & Marcos, M. Vertical land motion as a key to understanding sea level change and variability. *Rev. Geophys.* **54**, 64–92 (2016). 10.1002/2015RG000502

[3] Pfeffer, J., Spada, G., Mmin, A., Boy, J.-P. & Allemand, P. Decoding the origins of vertical land motions observed today at coasts. *Geophys. J. Int.* **210**, 148–165 (2017). 10.1093/gji/ggx142

[4] Hammond, W. C., Blewitt, G., Kreemer, C. & Nerem, R. S. GPS imaging of global vertical land motion for studies of sea level rise. *J. Geophys. Res.: Solid Earth* **126**, 2021–022355 (2021).

[5] Klos, A., Kusche, J., Fenoglio-Marc, L., Bos, M. S. & Bogusz, J. Introducing a vertical land motion model for improving estimates of sea level rates derived from tide gauge records affected by earthquakes. *GPS Solutions* **23**, 102 (2019).

[6] Frederikse, T., Landerer, F. W. & Caron, L. The imprints of contemporary mass redistribution on local sea level and vertical land motion observations. *Solid Earth* **10**, 1971–1987 (2019). 10.5194/se-10-1971-2019

[7] Ray, R., Loomis, B. & Zlotnicki, V. The mean seasonal cycle in relative sea level from satellite altimetry and gravimetry. *J. Geod.* **95**, 80 (2021). 34720451 10.1007/s00190-021-01529-1 PMC8550057

[8] Emery, K.O. & Aubrey, D.G. *Sea Levels, Land Levels, and Tide Gauges* (Springer, 1991); 10.1007/978-1-4613-9101-2

[9] Kolker, A.S., Allison, M.A. & Hameed, S. An evaluation of subsidence rates and sea-level variability in the northern Gulf of Mexico. *Geophys. Res. Lett.*10.1029/2011GL049458 (2011).

[10] Liu, Y., Li, J., Fasullo, J. & Galloway, D. L. Land subsidence contributions to relative sea level rise at tide gauge Galveston Pier 21, Texas. *Sci. Rep.* **10**, 17905 (2020). 33087790 10.1038/s41598-020-74696-4 PMC7578811

[11] Church, J. & White, N. Sea-level rise from the late 19th to the early 21st century. *Surv. Geophys.* **32**, 585–602 (2011). 10.1007/s10712-011-9119-1

[12] Hay, C. C., Morrow, E., Kopp, R. E. & Mitrovica, J. X. Probabilistic reanalysis of twentieth-century sea-level rise. *Nature*10.1038/nature14093 (1990). 10.1038/nature14093 25629092

[13] Dangendorf, S. et al. Persistent acceleration in global sea-level rise since the 1960s. *Nat. Clim. Change* **9**, 705–710 (2019). 10.1038/s41558-019-0531-8

[14] Oelsmann, J. et al. Bayesian modelling of piecewise trends and discontinuities to improve the estimation of coastal vertical land motion. *J. Geod.* **96**, 62 (2022). 10.1007/s00190-022-01645-6

[15] Slangen, A. B. A. et al. The evolution of 21st century sea-level projections from IPCC AR5 to AR6 and beyond. *Camb. Prisms: Coast. Futur.* **1**, e7 (2023).

[16] Slangen, A. B. A. et al. Projecting twenty-first century regional sea-level changes. *Climatic Change* **124**, 317–332 (2014). 10.1007/s10584-014-1080-9

[17] Jackson, L. P. & Jevrejeva, S. A probabilistic approach to 21st century regional sea-level projections using RCP and high-end scenarios. *Glob. Planet. Change* **146**, 179–189 (2016). 10.1016/j.gloplacha.2016.10.006

[18] Oppenheimer, M. et al. in *IPCC Special Report on the Ocean and Cryosphere in a Changing Climate* (eds Pörtner, H.-O. et al.) Ch. 4 (Cambridge Univ. Press, 2019).

[19] Kopp, R. E. et al. Probabilistic 21st and 22nd century sealevel projections at a global network of tidegauge sites. *Earth’s Future* **2**, 383–406 (2014). 10.1002/2014EF000239

[20] Husson, L., Bodin, T., Spada, G., Choblet, G. & Kreemer, C. Bayesian surface reconstruction of geodetic uplift rates: mapping the global fingerprint of Glacial Isostatic Adjustment. *J. Geodyn.* **122**, 25–40 (2018). 10.1016/j.jog.2018.10.002

[21] Hawkins, R., Husson, L., Choblet, G., Bodin, T. & Pfeffer, J. Virtual tide gauges for predicting relative sea level rise. *J. Geophys. Res.: Solid Earth* **124**, 13367–13391 (2019). 10.1029/2019JB017943

[22] Frederikse, T. et al. The causes of sea-level rise since 1900. *Nature* **584**, 393–397 (2020). 32814886 10.1038/s41586-020-2591-3

[23] Inman, D. L. & Nordstrom, C. E. On the tectonic and morphologic classification of coasts. *J. Geol.* **79**, 1–21 (1971). 10.1086/627583

[24] Pedoja, K. et al. Relative sea-level fall since the last interglacial stage: are coasts uplifting worldwide? *Earth Sci. Rev.* **108**, 1–15 (2011).

[25] Pedoja, K. et al. On the long-lasting sequences of coral reef terraces from SE Sulawesi (Indonesia): distribution, formation, and global significance. *Quat. Sci. Rev.* **188**, 37–57 (2018). 10.1016/j.quascirev.2018.03.033

[26] Vigny, C. et al. The 2010 Mw 8.8 Maule megathrust earthquake of central Chile, monitored by GPS. *Science* **332**, 1417–1421 (2011). 21527673 10.1126/science.1204132

[27] Imakiire, T. & Koarai, M. Wide-area land subsidence caused by the 2011 off the Pacific Coast of Tohoku earthquake. *Soils Found.* **52**, 842–855 (2012). 10.1016/j.sandf.2012.11.007

[28] Gunawan, E. et al. A comprehensive model of postseismic deformation of the 2004 Sumatra Andaman earthquake deduced from GPS observations in northern Sumatra. *J. Asian Earth Sci.* **88**, 218–229 (2014). 10.1016/j.jseaes.2014.03.016

[29] Houlié, N. & Stern, T. A. Vertical tectonics at an active continental margin. *Earth Planet. Sci. Lett.* **457**, 292–301 (2017). 10.1016/j.epsl.2016.10.018

[30] Naish, T. et al. The significance of vertical land movements at convergent plate boundaries in probabilistic sea-level projections for AR6 scenarios: the New Zealand case. *Earth Space Sci. Open Arch.*10.1002/essoar.10511878.1 (2022).

[31] Gómez, J. F., Kwoll, E., Walker, I. J. & Shirzaei, M. Vertical land motion as a driver of coastline changes on a deltaic system in the Colombian Caribbean. *Geosciences* **11**, 300 (2021). 10.3390/geosciences11070300

[32] Raucoules, D. et al. Remote sensing of environment high nonlinear urban ground motion in Manila (Philippines) from 1993 to 2010 observed by DInSAR: implications for sea-level measurement. *Remote Sens. Environ.* **139**, 386–397 (2013). 10.1016/j.rse.2013.08.021

[33] Buzzanga, B., Bekaert, D. P. S., Hamlington, B. D. & Sangha, S. S. Toward sustained monitoring of subsidence at the coast using INSAR and GPS: an application in Hampton Roads, Virginia. *Geophys. Res. Lett.*10.1029/2020GL090013 (2020).

[34] Syvitski, J. P. M. et al. Sinking deltas due to human activities. *Nat. Geosci.* **2**, 681–686 (2009). 10.1038/ngeo629

[35] Ericson, J., Vorosmarty, C., Dingman, S., Ward, L. & Meybeck, M. Effective sea-level rise and deltas: causes of change and human dimension implications. *Glob. Planet. Change* **50**, 63–82 (2006). 10.1016/j.gloplacha.2005.07.004

[36] Nicholls, R. J. et al. A global analysis of subsidence, relative sea-level change and coastal flood exposure. *Nat. Clim. Change* **11**, 338–342 (2021). 10.1038/s41558-021-00993-z

[37] Blewitt, G. & Kreemer, C. Harnessing the GPS data explosion for interdisciplinary science. *Eos*10.1029/2018EO104623 (2018).

[38] Kopp, R. E. Does the mid-Atlantic United States sea level acceleration hot spot reflect ocean dynamic variability?: sea level acceleration hot spot. *Geophys. Res. Lett.* **40**, 3981–3985 (2013). 10.1002/grl.50781

[39] Letetrel, C. et al. Estimation of vertical land movement rates along the coasts of the Gulf of Mexico over the past decades. *Cont. Shelf Res.* **111**, 42–51 (2015). 10.1016/j.csr.2015.10.018

[40] Riddell, A.R., King, M.A. & Watson, C.S. Present day vertical land motion of Australia from GPS observations and geophysical models. *J. Geophys. Res. Solid Earth*10.1029/2019JB018034 (2020).

[41] Piecuch, C. G. et al. Origin of spatial variation in US East Coast sea-level trends during 1900–2017. *Nature* **564**, 400–404 (2018). 10.1038/s41586-018-0787-6 30568196

[42] Karegar, M. A., Dixon, T. H., Malservisi, R., Kusche, J. & Engelhart, S. E. Nuisance flooding and relative sea-level rise: the importance of present day land motion. *Sci. Rep.* **7**, 11197 (2017). 28894195 10.1038/s41598-017-11544-y PMC5593944

[43] Karegar, M. A., Dixon, T. H. & Engelhart, S. E. Subsidence along the Atlantic coast of North America: Insights from GPS and late Holocene relative sea level data. *Geophys. Res. Lett.* **43**, 3126–3133 (2016). 10.1002/2016GL068015

[44] Caron, L. et al. GIA model statistics for grace hydrology, cryosphere, and ocean science. *Geophys. Res. Lett.* **45**, 2203–2212 (2018). 10.1002/2017GL076644

[45] Eyring, V. et al. Overview of the Coupled Model Intercomparison Project Phase 6 (CMIP6) experimental design and organization. *Geosci. Model Dev.* **9**, 1937–1958 (2016). 10.5194/gmd-9-1937-2016

[46] O’Neill, B. C. et al. The Scenario Model Intercomparison Project (ScenarioMIP) for CMIP6. *Geosci. Model Dev.* **9**, 3461–3482 (2016). 10.5194/gmd-9-3461-2016

[47] Kopp, R. E., et al.: The Framework for Assessing Changes To Sea-level (FACTS) v1.0: a platform for characterizing parametric and structural uncertainty in future global, relative, and extreme sea-level change. *Geosci*. *Model Dev.* **16**, 7461–7489 (2023).

[48] Garner, G. et al. IPCC AR6 sea level projections. *Zenodo*10.5281/zenodo.6382554 (2021).

[49] Geller, R. J. Earthquake prediction: a critical review. *Geophys. J. Int.* **131**, 425–450 (1997). 10.1111/j.1365-246X.1997.tb06588.x

[50] Kanamori, H. in *International Handbook of Earthquake and Engineering Seismology, Part B. International Geophysics* vol. 81 (eds Lee, W. H. K. et al.) 1205–1216 (Academic Press, 2003).

[51] Tay, C. et al. Sea-level rise from land subsidence in major coastal cities. *Nat. Sustain.*10.1038/s41893-022-00947-z (2022). 10.1038/s41893-022-00947-z

[52] Hamling, I. J., Wright, T. J., Hreinsdóttir, S. & Wallace, L. M. A snapshot of New Zealand’s dynamic deformation field from Envisat INSAR and GNSS observations between 2003 and 2011. *Geophys. Res. Lett.*10.1029/2021GL096465 (2022).

[53] Cazenave, A. et al. Sea level changes from Topex-Poseidon altimetry and tide gauges, and vertical crustal motions from DORIS. *Geophys. Res. Lett.*10.1029/1999GL900472 (1999). 10.1029/1999GL900472

[54] Nerem, R. S. & Mitchum, G. T. Estimates of vertical crustal motion derived from differences of TOPEX/POSEIDON and tide gauge sea level measurements. *Geophys. Res. Lett.*10.1029/2002gl015037 (2003). 10.1029/2002gl015037

[55] Kuo, C. Y., Shum, C. K., Braun, A. & Mitrovica, J.X. Vertical crustal motion determined by satellite altimetry and tide gauge data in Fennoscandia. *Geophys. Res. Lett.* 10.1029/2003GL019106 (2004).

[56] Pfeffer, J. & Allemand, P. The key role of vertical land motions in coastal sea level variations: a global synthesis of multisatellite altimetry, tide gauge data and GPS measurements. *Earth Planet. Sci. Lett.* **439**, 39–47 (2016). 10.1016/j.epsl.2016.01.027

[57] Kleinherenbrink, M., Riva, R. & Frederikse, T. A comparison of methods to estimate vertical land motion trends from GNSS and altimetry at tide gauge stations. *Ocean Sci.* **14**, 187–204 (2018). 10.5194/os-14-187-2018

[58] Santamaría-Gómez, A., Gravelle, M. & Wöppelmann, G. Long-term vertical land motion from double-differenced tide gauge and satellite altimetry data. *J. Geod.* **88**, 207–222 (2014). 10.1007/s00190-013-0677-5

[59] Rebischung, P., Altamimi, Z., Ray, J. & Garayt, B. The IGS contribution to ITRF2014. *J. Geod.* **90**, 611–630 (2016). 10.1007/s00190-016-0897-6

[60] Blewitt, G., Kreemer, C., Hammond, W. C. & Gazeaux, J. Midas robust trend estimator for accurate GPS station velocities without step detection. *J. Geophys. Res.: Solid Earth* **121**, 2054–2068 (2016). 27668140 10.1002/2015JB012552 PMC5024356

[61] Holgate, S.J. et al. New data systems and products at the permanent service for mean sea level. *J. Coast. Res*. **29**, 493–504 (2013).

[62] Carrère, L. & Lyard, F. Modeling the barotropic response of the global ocean to atmospheric wind and pressure forcing—comparisons with observations. *Geophys. Res. Lett.*10.1029/2002GL016473 (2003).

[63] Lyard, F. H., Allain, D. J., Cancet, M., Carr’ere, L. & Picot, N. Fes2014 global ocean tide atlas: design and performance. *Ocean Sci.* **17**, 615–649 (2021). 10.5194/os-17-615-2021

[64] Oelsmann, J. et al. The zone of influence: matching sea level variability from coastal altimetry and tide gauges for vertical land motion estimation. *Ocean Sci.* **17**, 35–57 (2021). 10.5194/os-17-35-2021

[65] Hay, C. C., Morrow, E. D., Kopp, R. E. & Mitrovica, J. X. On the robustness of Bayesian fingerprinting estimates of global sea level change. *J. Clim.* **30**, 3025–3038 (2017). 10.1175/JCLI-D-16-0271.1

[66] Riva, R., Frederikse, T., King, M., Marzeion, B. & Van den Broeke, M. Brief communication: the global signature of post-1900 land ice wastage on vertical land motion. *Cryosphere* **11**, 1327–1332 (2017). 10.5194/tc-11-1327-2017

[67] Tapley, B. D., Bettadpur, S., Watkins, M. & Reigber, C. The gravity recovery and climate experiment: mission overview and early results: GRACE mission overview and early results. *Geophys. Res. Lett*. 10.1029/2004GL019920 (2004).

[68] Kornfeld, R. P. et al. GRACE-FO: the Gravity Recovery and Climate Experiment Follow-On mission. *J. Spacecraft Rockets* **56**, 931–951 (2019). 10.2514/1.A34326

[69] Wudong, L. et al. Extracting common mode errors of regional GNSS position time series in the presence of missing data by variational Bayesian principal component analysis. *Sensors* **20**, 2298 (2020). 32316478 10.3390/s20082298 PMC7219079

[70] Gazeaux, J. et al. Detecting offsets in GPS time series: first results from the detection of offsets in GPS experiment. *J. Geophys. Res.: Solid Earth* **118**, 2397–2407 (2013). 10.1002/jgrb.50152

[71] Gruszczynski, M., Klos, A. & Bogusz, J. A filtering of incomplete GNSS position time series with probabilistic principal component analysis. *Pure Appl. Geophys.* **175**, 1841–1867 (2018). 10.1007/s00024-018-1856-3

[72] Hoffman, M. D. & Gelman, A. The no-u-turn sampler: adaptively setting path lengths in Hamiltonian Monte Carlo. *J. Mach. Learn. Res.* **15**, 1593–1623 (2014).

[73] Gelman, A. & Rubin, D. B. Inference from iterative simulation using multiple sequences. *Stat. Sci.* **7**, 457–472 (1992). 10.1214/ss/1177011136

[74] Vehtari, A., Gelman, A., Simpson, D., Carpenter, B. & Bürkner, P.-C. Rank-normalization, folding, and localization: an improved R for assessing convergence of MCMC (with discussion). *Bayesian Anal.* **16**, 667–718 (2021). 10.1214/20-BA1221

[75] Gelman, A. et al. *Bayesian Data Analysis* (Chapman and Hall/CRC, 2020); http://www.stat.columbia.edu/~gelman/book/

[76] Bodin, T., Salmon, M., Kennett, B. L. N. & Sambridge, M. Probabilistic surface reconstruction from multiple data sets: an example for the Australian moho. *J. Geophys. Res.: Solid Earth*10.1029/2012JB009547 (2012).

[77] Hawkins, R., Bodin, T., Sambridge, M., Choblet, G. & Husson, L. Trans-dimensional surface reconstruction with different classes of parameterization. *Geochem. Geophys. Geosyst.* **20**, 505–529 (2019). 10.1029/2018GC008022

[78] Bodin, T. & Sambridge, M. Seismic tomography with the reversible jump algorithm. *Geophys. J. Int.* **178**, 1411–1436 (2009). 10.1111/j.1365-246X.2009.04226.x

[79] Mosegaard, K. & Tarantola, A. Monte Carlo sampling of solutions to inverse problems. *J. Geophys. Res.: Solid Earth* **100**, 12431–12447 (1995). 10.1029/94JB03097

[80] Sambridge, M. & Mosegaard, K. Monte Carlo methods in geophysical inverse problems. *Rev. Geophys.* **40**, 3–1329 (2002). 10.1029/2000RG000089

[81] Newton, T. et al. An assessment of vertical land movement to support coastal hazards planning in Washington state. *Water* **13**, 281 (2021). 10.3390/w13030281

[82] James, T. S., Gowan, E. J., Wada, I. & Wang, K. Viscosity of the asthenosphere from glacial isostatic adjustment and subduction dynamics at the northern Cascadia subduction zone, British Columbia, Canada. *J. Geophys. Res.* **114**, 04405 (2009). 10.1029/2008JB006077

[83] Engelhart, S. E., Vacchi, M., Horton, B. P., Nelson, A. R. & Kopp, R. E. A sea-level database for the Pacific coast of central North America. *Quat. Sci. Rev.* **113**, 78–92 (2015). 10.1016/j.quascirev.2014.12.001

[84] Yousefi, M., Milne, G. A., Love, R. & Tarasov, L. Glacial isostatic adjustment along the Pacific coast of central North America. *Quat. Sci. Rev.* **193**, 288–311 (2018). 10.1016/j.quascirev.2018.06.017

[85] Emery, K. O. & Aubrey, D. G. in *Sea Levels, Land Levels, and Tide Gauges* 23–52 (Springer, 1991); 10.1007/978-1-4613-9101-2

[86] Wessel, P. & Smith, W. H. F. A global, self-consistent, hierarchical, high-resolution shoreline database. *J. Geophys. Res.: Solid Earth* **101**, 8741–8743 (1996). 10.1029/96JB00104

