# Supplementary material for: Regional variations in relative sea-level changes influenced by nonlinear vertical land motion
Source: Nat Geosci. 2024 Feb 12;17(2):137–44. doi: 10.1038/s41561-023-01357-2 (PMC11371649; doi:10.1038/s41561-023-01357-2)
Supplement: Supplementary file 1 — Supplementary Methods. [file 41561_2023_1357_MOESM1_ESM.pdf]

nature geoscience

Article https://doi.org/10.1038/s41561-023-01357-2

Regional variations in relative sea-level changes influenced by nonlinear vertical land motion

In the format provided by the authors and unedited

Supplementary Methods

Vertical land motion reconstruction unveils non-linear effects on relative sea level changes from 1900-2150

Julius Oelsmann, Marta Marcos, Marcello Passaro, Laura Sanchez, Denise Dettmering, Sönke Dangendorf and Florian Seitz

---

Synthetic experiments

Here we provide an additional validation of our approach (Bayesian Principal Component Analysis, BPCA) using synthetic data. Our goal is to estimate the true underlying parameters of the following model $U(x,t)$:

$$U(x,t) = g(x)\,t + W(x)\,PC(t) + \sigma^2_{GNSS,\ SATTG}$$

Component		
Trends	$g(x) = N(\widehat{g(x)}, \sigma_g^2)$	$\sigma_g = 0.5\ mm/y$
Pattern (1. EOF)	$W(x) = N(\widehat{W(x)}, \sigma_W^2)$	$\sigma_W = 0.5$
Principal component	$PC(t)$	
Instrumental errors (white noise)	$\sigma^2_{GNSS,\ SATTG}$	$\sigma_{GNSS} = 5\ mm$<br>$\sigma_{SATTG} = 15\ mm$

The hat symbol indicates the true parameter value. σ_g^2 and σ_W^2 represent the amplitudes of normally distributed perturbations in space, $\sigma^2_{GNSS,\ SATTG}$ is white noise added to the model (after applying σ_g^2 and σ_W^2). All values reflect realistic amplitudes based on the analysis of the GNSS and SATTG data used in the paper. We consider annual averages and a period of 26 years. Our background pattern of the trends and the first mode are generated using superimposed 2D Gaussian functions.

Experiment 1:

In our first experiment we will estimate the parameters of the model associated with the values shown in Figure 1.

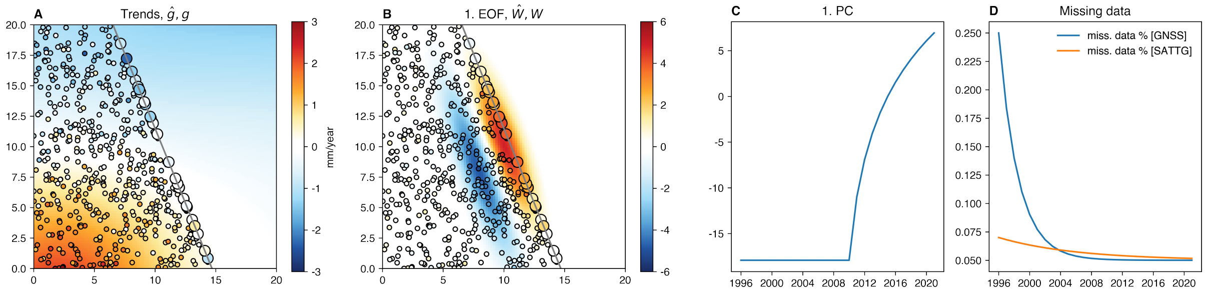

*Figure 1: Synthetic data: **A**: True trends $\widehat{g(x)}$ and perturbed point estimates $\widehat{g(x)} + \sigma_g^2$. **B**: True pattern of the first mode of variability $\widehat{W(x)}$ and perturbed point estimates $\widehat{W(x)} + \sigma_W^2$. **C**: Associated time series and (**D**) fraction of missing data per observation type. Here the line represents an arbitrary coastline, where tide gauges are located (as indicated by the larger marker sizes in the scatter plot). The location of the data is randomly chosen.*

The true model parameters $\widehat{g(x)}$ and $\widehat{W(x)}$ are indicated by the continuous surfaces in the background. These parameters are perturbed (as explained above) to mimic some variability across individual stations (as shown by the scattered data). Here, the larger (smaller) circles represent 30 tide gauge stations (500 GNSS stations). We also simulate some time-dependent missing data, as shown in Figure 1D.

Results:

We fit the data using the BPCA model (as described in the paper), including a single mode of variability, and using 1500 iterations after 2000 tuning steps.

To test whether the chains have converged we consider the potential scale reduction factor $\hat{R}$ and the relative effective sample site ESS per iteration n. $\hat{R}$ is a widely used convergence parameter which provides a measure of the standard deviation across chains, versus the within-chain variability. When $\hat{R}$ does not converge to one it indicates that the chains have likely not converged to the equilibrium distribution (Gelman and Rubin 1992, Gelman et al., 2020).

We obtain the following averaged (over all dimensions) model diagnostics:

	**PC(t)**	**W(x)**	**g(x)**	σ
**ESS/n**	0.066	0.71	1.826	2.042
$\hat{R}$	1.040	1.006	1.000	1.000

Here, $\hat{R}$ is < 1.05 for the considered parameters which indicates good convergence.

Figure 2 exemplifies some of the model estimates for individual stations (which are randomly selected). The prescribed data is shown in orange (and with white noise $\sigma^2_{GNSS,\ SATTG}$ in red) and the model estimate (and 1σ uncertainties) are shown in blue. The uncertainties are computed by combining all individual uncertainties (i.e., the standard deviations of g(x), W(x), and PC(t)). We observe a good recovery of the underlying model, despite the white noise perturbations.

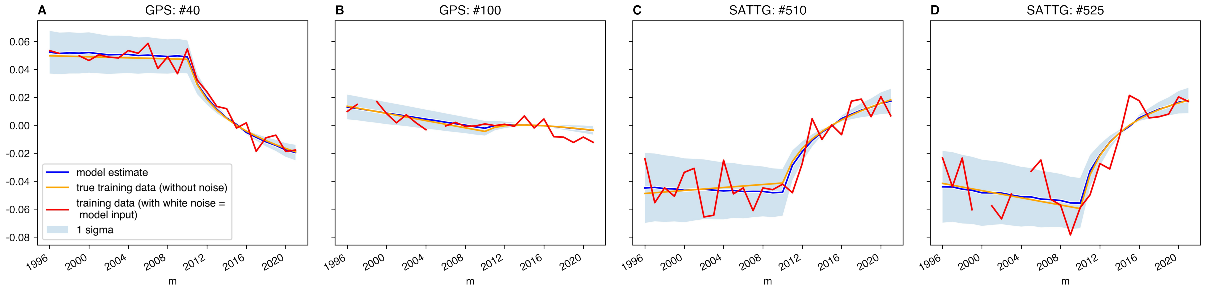

*Figure 2: Individual time series fits, for GNSS (**A,B**) and SATTG (**C,D**). Shown are the prescribed model **U** in orange (and with white noise in red), the estimate in blue, and the 1σ uncertainty of the model estimate in blue shadings.*

Figure 3 presents the model results much more comprehensively. Here, Figures 3 **E** and **F** show the BPCA estimates of the perturbed model parameters $g(x)$ and $W(x)$. These parameters, together with the estimate of the time series (shown in Figure 3 **G** with 1σ uncertainties) indicate a accurate model performance. The quality of the fit is quantified using the significance ratio of the differences of the BPCA estimates and the prescribed perturbed model parameters $g(x)$ and $W(x)$ and the combined uncertainties of the estimates and the prescribed parameters. While the uncertainties of the model estimates correspond to the standard deviation computed over the samples of the Markov chains of the estimated model, the prescribed model parameter uncertainties (i.e., the standard errors of the parameters) are computed analytically based on knowledge of the model residuals, which is equivalent to the white noise perturbations. Note, that we also keep the time series $PC(t)$ as a constant term in

the design matrix, when computing these uncertainties analytically. Figures 3 **K** and **L** indicate that for both time series types (SATTG and GNSS data), and both model parameters $g(x)$ and $W(x)$, the significance ratios are mostly between ± 1, indicating that the parameters are generally well recovered within the estimated uncertainties. Figure 3 **H** substantiates that also the white noise amplitudes $\sigma^2_{\mathrm{GPS,\ SATTG}}$ of the different time series types are estimated accurately.

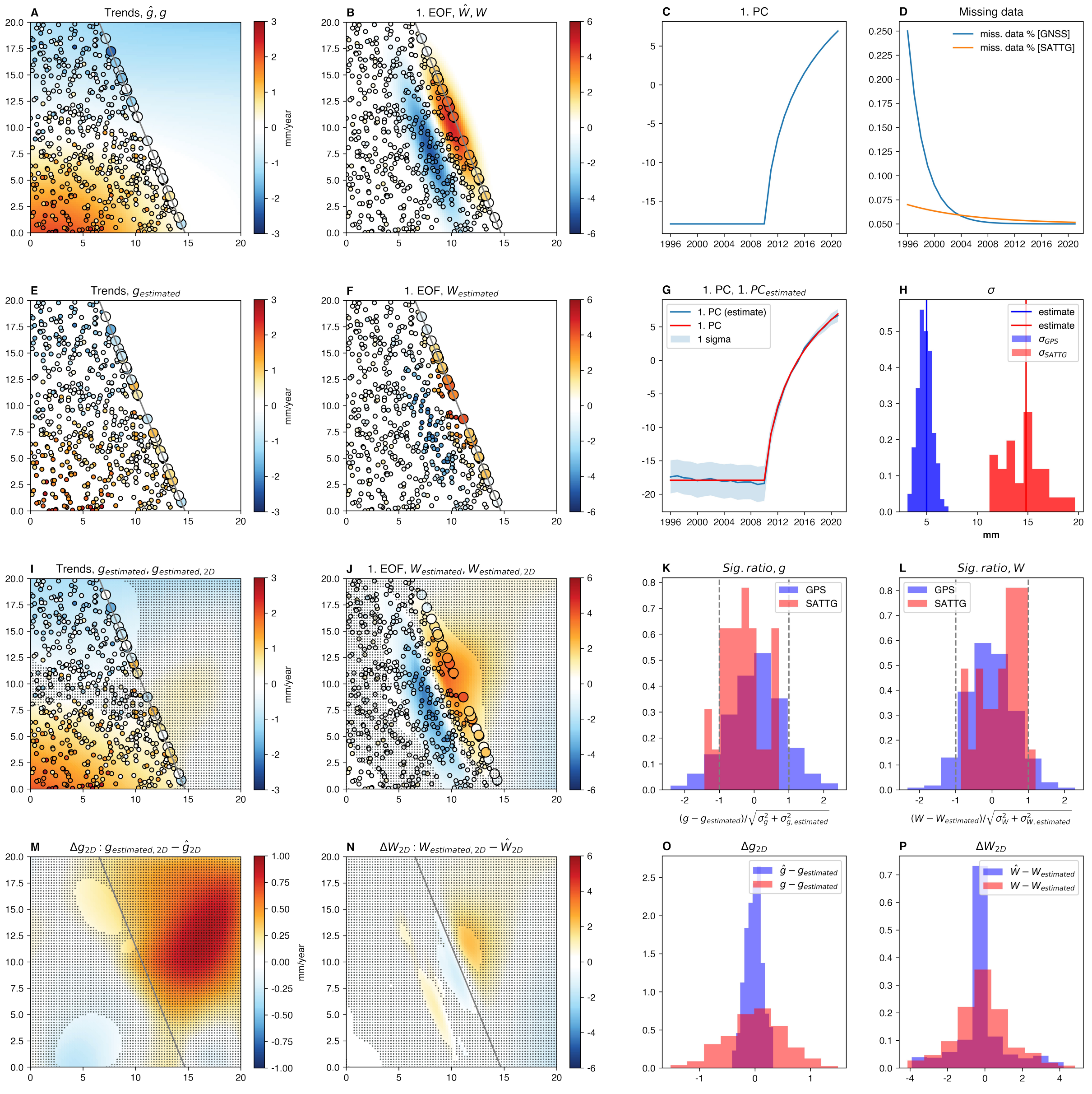

Figure 3: Synthetic data: **A**: True trends $\widehat{g(x)}$ and perturbed point estimates $\widehat{g(x)} + \sigma_g^2$. **B**: True pattern of the first mode of variability $\widehat{W(x)}$ and perturbed point estimates $\widehat{W(x)} + \sigma_W^2$. **C**: Associated time series and (**D**) fraction of missing data per observation type. Here the line represents an arbitrary coastline, where tide gauges are located (as indicated by the larger marker sizes in the scatter plot). The location of the data is randomly chosen. **E** and **F**, estimates of the perturbed model parameters $g(x)$ and $W(x)$. **G** shows the time series of the first mode and the model estimate (with 1σ uncertainties). **H** shows the prescribed amplitudes $\sigma^2_{GNSS,\ SATTG}$ of the added white noise (i.e. time series noise), and the model mean estimate of these amplitudes (vertical lines). **I** and **J** show the point estimates of $g(x)$ and $W(x)$ (as in E and F), together with the interpolated values obtained after Transdimensional regression. Stipplings indicate significant values, i.e., values (deviations from zero) greater than their uncertainties. **K** and **L** show the significance ratios of the prescribed and the estimated parameters $g(x)$ and $W(x)$ (which also accounts for their uncertainties). **M** and **N** show the differences of the interpolated model parameters and the true underlying model (excluding the perturbations in space, as applied in A and B). Positive values indicate here overestimation, and vice versa. Stipplings indicate regions where these differences are significant, by only accounting for the uncertainties of the interpolation and not accounting for any uncertainty of the true model parameters $\widehat{g(x)}$, $\widehat{W(x)}$. **O** and **P** show the normalized histograms of the differences of the 2D interpolated estimates and the true model parameters $\widehat{g(x)}$, $\widehat{W(x)}$ in blue, and the perturbed parameters $g(x)$, $W(x)$, which are actually used as input parameters.

After the point-wise estimation of the model parameters, we apply the Bayesian transdimensional regression to obtain 2D interpolated surfaces. The interpolated values are shown in Figures 3 **I** and **J** together with the point estimates of $g(x)$ and $W(x)$ (as in **E** and **F**). The stipplings indicate significant values, that is, any absolute values that are greater than their uncertainties (in this case 2σ). Note, that for this and the following plots we do not apply any field significance checks. This plot illustrates, that in regions with no prescribed observations (especially in the right half of the domain), the 2D interpolation may extrapolate some of the values, which are, however, generally smaller than their associated estimated uncertainties. Thus, this analysis supports that our approach does not generate significant model estimates in areas where we have limited or no knowledge of the true model from observations.

In Figures 3 **M** and **N** we show the differences of the interpolated model parameters and the true underlying model ($\widehat{g(x)}$and $\widehat{W(x)}$, excluding the perturbations in space, as applied in Figures 3 A and B). Positive values indicate overestimation of the model parameters, and vice versa. Again, we use stipplings to indicate regions where these differences are significant. However, we only account for the uncertainties obtained from the Transdimensional regression and not for any uncertainties of the true model parameters $\widehat{g(x)}$, $\widehat{W(x)}$ in 2D (which are hard to quantify, mainly because the 2D model uncertainties are a function of the spatial perturbations σ_g^2 and σ_W^2, as well as the added white noise). Even without accounting for any uncertainty of the true underlying model, the differences of the estimated and the prescribed parameters are generally insignificant (considering 2σ uncertainties). This indicates an overall good recovery of the true underlying model parameters $\widehat{g(x)}$, $\widehat{W(x)}$. The differences between the 2D interpolated parameters and the true model parameters $\widehat{g(x)}$, $\widehat{W(x)}$ and the perturbed parameters $g(x)$, $W(x)$, respectively, are shown in Figures 3 **O** and **P** and for the individual locations. For both parameters we see that the true underlying parameters $\widehat{g(x)}$, $\widehat{W(x)}$ are much better recovered than the spatially perturbed parameters. This is mostly caused by the spatial interpolation, which averages out some of the spatial variability. Overall, this result highlights the value of the network-based approach to avoid spurious location dependent variability, or noise and to recover the underlying variability.

Experiment 2: Spatial data gaps

In this experiment we investigate the model performance if some of the underlying observations are omitted from the dataset. We use the exact same data as before and omit all data between the 10th and 13th coordinate of the y-dimension (resulting in 458 remaining stations). We perform the exact same steps (model runs, model validation) as before. The data and the validation results are shown in Figure 4.

Qualitatively, and in most cases quantitatively, the estimation is very similar to the previous experiment, as confirmed by panels E to P of Figure 4. Interestingly, after the 2D interpolation, the model also recovers much of the underlying spatial structure of the 1. mode of variability, without any observational constraints within the spatial gap. This is likely facilitated by the ensemble of unstructured grid realizations, where individual grid cells can connect the regions across the domain where observations are missing. The Transdimensional regression does not require any a'priori information of the spatial covariance of the data, and can therefore adapt to the underlying data structure. Accordingly, the recovery of the true model parameters $\widehat{g(x)}$and $\widehat{W(x)}$ (as shown in Figures 4 **M** and **N**) is at least as good as for the experiment 1 (without observational gaps, as shown in Figures 3 **M** and **N**).

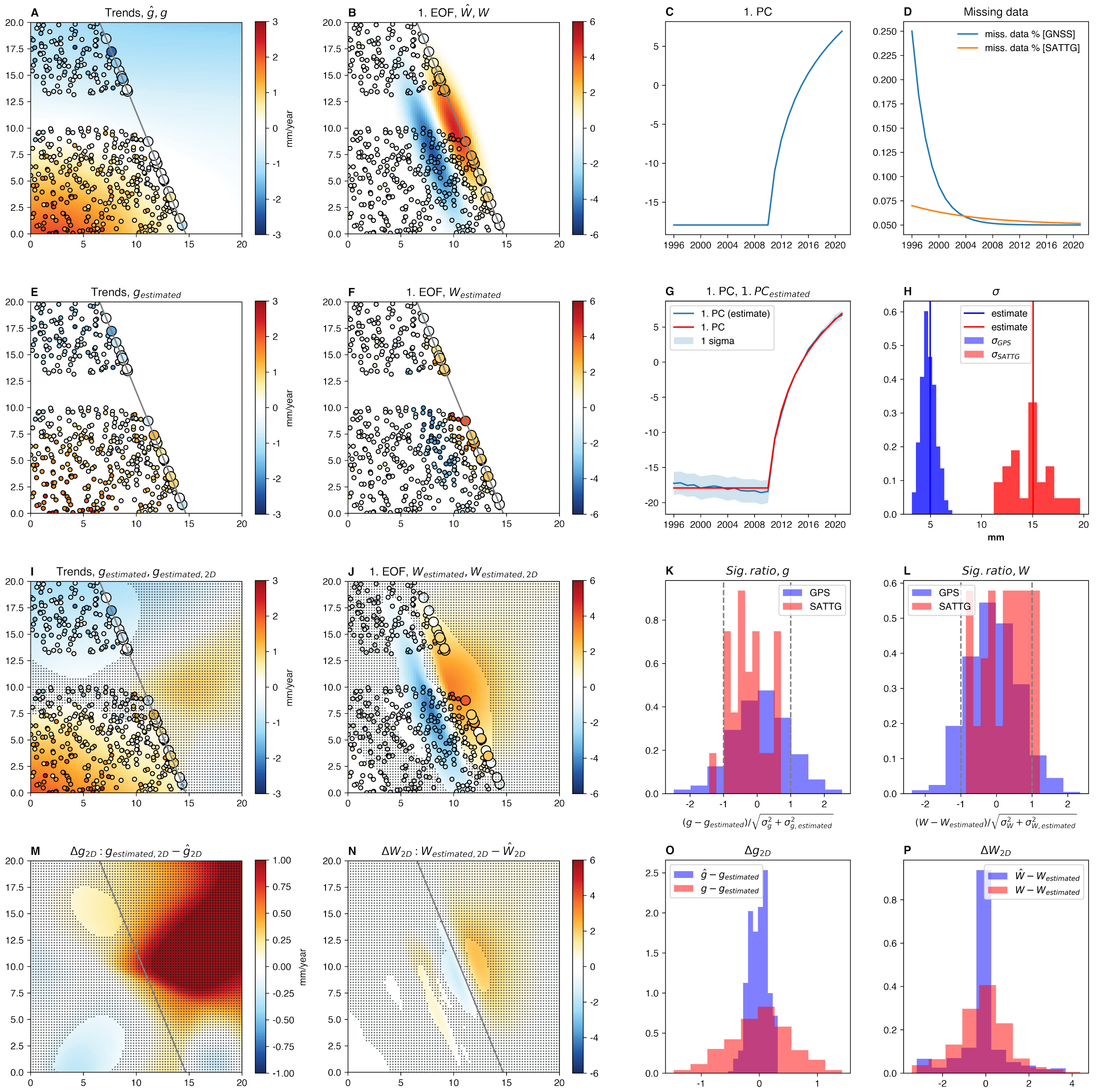

Figure 4: Synthetic data of the second experiment: ***A****: True trends $\widehat{g(x)}$ and perturbed point estimates $\widehat{g(x)} + \sigma_g^2$.* ***B****: True pattern of the first mode of variability $\widehat{W(x)}$ and perturbed point estimates $\widehat{W(x)} + \sigma_W^2$.* ***C****: Associated time series and (****D****) fraction of missing data per observation type. Here the line represents an arbitrary coastline, where tide gauges are located (as indicated by the larger marker sizes in the scatter plot). The location of the data is randomly chosen.* ***E*** *and* ***F****, estimates of the perturbed model parameters $g(x)$ and $W(x)$.* ***G*** *shows the time series of the first mode and the model estimate (with 1σ uncertainties).* ***H*** *shows the prescribed amplitudes $\sigma^2_{GNSS,\ SATTG}$ of the added white noise (i.e. time series noise), and the model mean estimate of these amplitudes (vertical lines).* ***I*** *and* ***J*** *show the point estimates of $g(x)$ and $W(x)$ (as in E and F), together with the interpolated values obtained after Transdimensional regression. Stipplings indicate significant values, i.e., values (deviations from zero) greater than their uncertainties.* ***K*** *and* ***L*** *show the significance ratios of the prescribed and the estimated parameters $g(x)$ and $W(x)$ (which also accounts for their uncertainties).* ***M*** *and* ***N*** *show the differences of the interpolated model parameters and the true underlying model (excluding the perturbations in space, as applied in A and B). Positive values indicate here overestimation, and vice versa. Stipplings indicate regions where these differences are significant, by only accounting for the uncertainties of the interpolation and not accounting for any uncertainty of the true model parameters $\widehat{g(x)}$, $\widehat{W(x)}$.* ***O*** *and* ***P*** *show the normalized histograms of the differences of the 2D interpolated estimates and the true model parameters $\widehat{g(x)}$, $\widehat{W(x)}$ in blue, and the perturbed parameters $g(x)$, $W(x)$, which are actually used as input parameters.*

References:

Gelman, A., Rubin, D.B.: Inference from Iterative Simulation Using Multiple Sequences. Statistical Science 7(4), 457{472 (1992). https://doi.org/10.1214/ss/1177011136

Gelman, A., Carlin, J., Stern, H., Dunson, D., Vehtari, A., Rubin, D.: Bayesian Data Analysis, (2020). http://www.stat.columbia.edu/~gelman/book/
